# Structure-Function Characterisation of Eop1 Effectors from the *Erwinia-Pantoea* Clade Reveals They May Acetylate Their Defence Target through a Catalytic Dyad

**DOI:** 10.3390/ijms241914664

**Published:** 2023-09-28

**Authors:** Vishant Tomar, Erik H. A. Rikkerink, Janghoon Song, Svetla Sofkova-Bobcheva, Vincent G. M. Bus

**Affiliations:** 1 Mt Albert Research Centre, The New Zealand Institute for Plant and Food Research Limited, Auckland 1025, New Zealand; 2 School of Agriculture and Environment, Massey University, Private Bag 11222, Palmerston North 4442, New Zealand; s.sofkova@massey.ac.nz; 3 Pear Research Institute, National Institute of Horticultural & Herbal Science, Rural Development Administration, Naju 58216, Republic of Korea; 4 Hawkes Bay Research Centre, The New Zealand Institute for Plant and Food Research Limited, Havelock North 4130, New Zealand; vincent.bus@plantandfood.co.nz

**Keywords:** fire blight, Eop1, YopJ, catalytic dyad, AlphaFold models, acetylation, *Erwinia amylovora*, effector

## Abstract

The YopJ group of acetylating effectors from phytopathogens of the genera *Pseudomonas* and *Ralstonia* have been widely studied to understand how they modify and suppress their host defence targets. In contrast, studies on a related group of effectors, the Eop1 group, lag far behind. Members of the Eop1 group are widely present in the *Erwinia-Pantoea* clade of Gram-negative bacteria, which contains phytopathogens, non-pathogens and potential biocontrol agents, implying that they may play an important role in agroecological or pathological adaptations. The lack of research in this group of YopJ effectors has left a significant knowledge gap in their functioning and role. For the first time, we perform a comparative analysis combining AlphaFold modelling, in planta transient expressions and targeted mutational analyses of the Eop1 group effectors from the *Erwinia-Pantoea* clade, to help elucidate their likely activity and mechanism(s). This integrated study revealed several new findings, including putative binding sites for inositol hexakisphosphate and acetyl coenzyme A and newly postulated target-binding domains, and raises questions about whether these effectors function through a catalytic triad mechanism. The results imply that some Eop1s may use a catalytic dyad acetylation mechanism that we found could be promoted by the electronegative environment around the active site.

## 1. Introduction

Self-defence against biotic stressors such as bacteria, fungi and animals is crucial for the survival of all biological organisms, including plants. However, being sessile, plants cannot avoid these stressors via locomotion. Consequently, plants have evolved with an intricate defence system that extends beyond the plants’ physical barriers, equipping every plant cell with the ability to perform complex immune activities to execute self-defence. Fire blight is a particularly damaging bacterial disease of several commercially significant Rosaceae species such as apple and pear, and the causal agent *Erwinia amylovora* secretes proteins into plant cells to suppress defence responses. Research to understand how these proteins modify their host targets is of significant scientific and commercial interest.

The plant immune system is generally classified into two tiers, namely (a) pattern-triggered immunity (PTI) and (b) effector-triggered immunity (ETI) [1]. PTI is the first tier of active defence against microbes and relies on a set of transmembrane proteins known as pattern recognition receptors (PRRs) [2,3,4]. ETI, the second tier of plant immunity, is often triggered by nucleotide-binding leucine-rich repeat (NLR) resistance proteins (R-proteins) [5,6,7].

During bacterial infection, PRRs activate immune signalling while adapted bacterial pathogens suppress PTI by delivering effector molecules into the cells of the host plant. These effectors promote infection by subverting the host’s immune system, e.g., by inducing post-translational modifications to targeted host proteins, resulting in effector-triggered susceptibility (ETS). To combat ETS, resistant plants employ R-proteins (such as NLRs) that perceive pathogen effectors and trigger ETI, the more robust form of plant immunity. Pathogens, in turn, respond to ETI by introducing novel effectors or novel mutations in the pre-existing effectors to escape recognition, which again results in ETS. This dynamic interaction between microbial pathogens and their corresponding hosts underpins co-evolution between the host defence system and the pathogen defence-suppressing machinery, determining host resistance or susceptibility in response to any pathogen invasion.

*Yersinia* outer protein J (YopJ) is a superfamily of evolutionarily conserved bacterial protein effectors whose members are found in animal and plant pathogens and symbionts [8,9,10]. The YopJ effectors were initially assumed to have a cysteine protease-like activity because of their structural and topological similarity to members of the C55 family of cysteine proteases, such as ubiquitin-like protease 1 (ULP1) and adenoviral protease (AVP) [10]. However, several YopJ effectors, namely PopP2 [11,12], AvrBsT [13], HopZ1a [14,15,16,17] and HopZ3 [18] were later discovered to modify their host targets post-translationally via acetylation [9].

The YopJ family effectors are distinguished from other acetyltransferases by a unique activation and function mechanism. Unlike other acetyltransferases, YopJ effectors require a eukaryote-specific co-factor, inositol hexakisphosphate (IP6), for activation [19]. IP6 activates the YopJ effector by inducing a conformational change, forming an “acetyl coenzyme A-binding pocket” adjacent to the catalytic motif [9,20]. The binding of acetyl coenzyme A (AcCoA) to the YopJ effector is a crucial step for its enzymatic activity as it provides the “acetyl” functional group for the process of acetylation [9]. The most widely accepted catalytic mechanism of the YopJ effectors is currently explained by the “ping-pong” model [9,21]. The model proposes a two-step mechanism which involves “autoacetylation” of the effector and “trans-acetylation” of its corresponding substrates [9,13,14,15,19,20,22]. The catalytic activity of the YopJ effectors reportedly relies on an evolutionarily conserved “catalytic triad”, typically comprised of three amino acid residues: histidine (H), glutamic acid (E), and cysteine (C) [9,10].

The “*Erwinia*-*Pantoea*” clade, comprised of Enterobacteriaceae members, includes several economically important, phytopathogenic and non-phytopathogenic species, such as *E. amylovora*, *E. pyrifoliae*, *E. tracheiphila*, *E. piriflorinigrans*, *E. tasmaniensis*, *E. billingiae*, *P. vagans* and *P. agglomerans* (refer to Adeolu et al. [23] and Janda and Abbott [24] for a comprehensive overview of this clade). YopJ-like effectors known as *Erwinia* outer protein 1 (Eop1) are widely present in this clade [25,26]; however, there has been no detailed analysis of their catalytic and enzymatic activity, leaving a significant knowledge gap in the functioning and role of these effectors. Sequence variation between the Eop1 effectors in this clade and YopJ effectors from other pathogens provides a unique opportunity to assess the basis of their respective catalytic mechanism(s). In this paper, we present a combination of sequence comparisons, structural modelling and targeted mutational analyses of the Eop1 effectors, and provide the first evidence for the likely enzymatic function and catalytic mechanism of Eop1 effectors from the *Erwinia-Pantoea* clade.

## 2. Results and Discussion

### 2.1. YopJ Family Effectors from E. amylovora and Other Related Species from the “Erwinia-Pantoea” Clade Trigger HR-like Cell Death in Nicotiana tabacum

When introduced into host plants, pathogen-delivered effectors act as virulence factors that assist the pathogen to successfully invade the host plant by modulating its innate immune system [27,28]. Paradoxically, when delivered into certain host genetic backgrounds, the same effectors can serve as avirulence factors and trigger a hypersensitive response (HR) through specific recognition via NLR-type immune receptors, imparting host resistance [27,29]. The effectors HopZ5, HopZ3 and HopZ1a from *Pseudomonas syringae* pathovars are classic examples of YopJ family avirulence factors, as they trigger HR-like cell death when introduced or expressed in *Nicotiana benthamiana* [30,31], *Nicotiana tabacum* [32] and *Arabidopsis thaliana* [20,31], respectively. These *P. syringae* effectors are sequence homologs of the *Erwinia*-*Pantoea* clade YopJs—Eop1 [9,33]. It was discovered previously that the phylogenetically related YopJ effector HopZ3 [34] triggers an HR when expressed transiently in *N. tabacum* [32], so we set out to test, for the first time, if the *Erwinia*-*Pantoea* clade Eop1 family effectors also trigger HR-like cell death in *N. tabacum*, reasoning that we could use this response to further characterise this understudied effector group.

Members of the YopJ superfamily effectors from *E. amylovora* and other related species from the “*Erwinia*-*Pantoea*” clade (hereafter referred to as “Eop1 variants”) were selected and tested via transient expression in the non-host plant *Nicotiana tabacum* “Samsun”. Protein variants for the analysis were selected based on differences in the protein sequence identity between 55 and 82% (Supplementary Figure S1) and the host plants associated with their strains of origin (Supplementary Table S1). The primary objective of this strategy was to include a diverse set of Eop1 variants from the plant-pathogenic or plant-associated (non-pathogens and epiphytes) species, while excluding those from an animal-pathogenic background, such as Eop1s of the *Yersinia* genus. Six Eop1 variants, along with HopZ3Psy (WP_003378257.1), a known trigger of HR in *N. tabacum* [32], were selected for the transient expression analysis (refer to Supplementary Table S1 for the information on the corresponding species and abbreviations used for the Eop1 variants selected for the HR assay).

**Figure 1:**
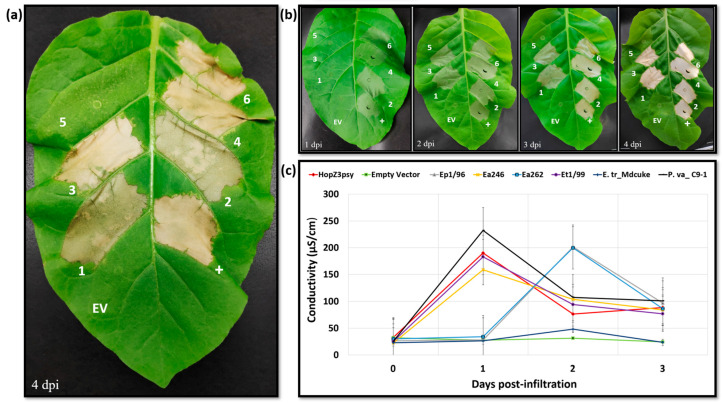
Transient expression analysis of the *Erwinia-Pantoea* clade Eop1 variants in *Nicotiana tabacum* “Samsun”: (**a**) Hypersensitive response-induced cell death phenotype observed in *N. tabacum* infiltrated with *Agrobacterium* harbouring the gene for Eop1 variants and HopZ3 effectors; (**b**) temporal profile of HR elicitation with the expression of *eop1* variants in *N. tabacum*; (**c**) electrolyte leakage assay from leaf discs obtained from *eop1* variant-infiltrated *N. tabacum* leaves. The numeric annotations in the figures on the leaf segments are as follows: “+”, HopZ3*Psy* (positive control); EV, empty vector; 1, *E. pyrifoliae* str. *Ep*1/96 Eop1; 2, *E. amylovora* str. *Ea*246 Eop1; 3, *E. amylovora* str. *Ea*262 Eop1; 4, *E. tasmaniensis* str. *Et*1/99 Eop1; 5, *E. tracheiphila* str. MDcuke Eop1; 6, *P. vagans* str. C9-1 Eop1. The experiments were repeated 5 times with similar results. The images were taken on the days indicated on the bottom left of the leaf images. The leaf width at 4 dpi was approximately 7 cm.

*Agrobacterium* cells carrying an expression clone harbouring the *eop1* gene variant under the 35S promoter were infiltrated into the leaves of 3–3.5 weeks old *N. tabacum* “Samsun” seedlings. HR-like cell death in the plant leaf, characterised by rapid necrosis followed by gradual mummification of the infiltrated leaf segment, was observed in five out of six tested Eop1 variants (Figure 1a). However, a difference in the time of HR elicitation was observed between different variants (Figure 1b). Three Eop1 variants, namely *Ea*246, *Et*1/99 and *P. va*_C9-1, together with the positive control, i.e., HopZ3psy, elicited a strong HR in their respective infiltrated regions at 1 day post-infiltration (dpi). Two Eop1 variants from *Ep*1/96 and *Ea*262 triggered HR at 2 dpi. Contrastingly, neither cell death nor any other phenotypic response was observed in the leaf segments infiltrated with *Agrobacterium* carrying the *E. tr*_MDcuke Eop1 expressing clone. To corroborate the observed phenotypes, an electrolyte leakage assay was also performed. The trend in conductivities paralleled the visual development of the HR phenotype. Eop1 variants from *Ea*246, *Et*1/99 and *P. va*_C9-1, and HopZ3Psy exhibited significant ion leakage at 1 dpi, with a mean peak value of more than 150 μS/cm (Figure 1c). Ep1/96 and Ea262 Eop1 variants exhibited the same HR trend at the delayed time of 2 dpi. On the other hand, no significant ion leakage, with a peak value of less than 50 μS/cm, was observed when using the *E. tr_*Mdcuke Eop1 variant. (Figure 1c).

Taken together, the findings suggest that the *Erwinia*-*Pantoea* clade Eop1 variants function as putative “avirulence factors” in the non-host plant *Nicotiana tabacum* “Samsun”. The results also suggest the presence of at least one R-protein, which could be driving the effectors’ molecular recognition and triggering the HR. The ability of the *E. tr_*Mdcuke Eop1 variant to escape molecular recognition provides an interesting contrast. The pathogen *E. tracheiphila* is known to trigger HR in *N. tabacum* [35]; however, the result presented in the current study suggests that (unlike Eop1 from *E. amylovora*, *E. tasmaniensis* and *E. pyrifoliae*) the *E. tracheiphila* Eop1 variant may not be able to trigger HR in tobacco. A recent study by Olawole et al. [33] suggested that minor sequence differences among Eop1-family effectors could influence their impacts on plant hosts, perhaps explaining why the Eop1 from *E. tr*_Mdcuke escapes molecular recognition. The same study found that *E. tracheiphila* Eop1s (unlike *E. amylovora* Eop1) did not contribute to host specificity but did contribute to host virulence on specific hosts. The *E. tr_*Mdcuke allele has been identified as increasing the virulence of strains isolated from squash on muskmelon, suggesting that the *E. tr_*Mdcuke allele has particular functional attributes. Although the Eop1 variants were all expressed in the same transient expression vector with the same promoter and infiltrated with equal volumes of Agrobacterium cells, we cannot completely rule out the possibility that expression differences may partly be contributing to differences in the visual HR symptoms. The ability of most of the tested Eop1 variants to trigger an HR in *N. tabacum*, and the phylogenetic similarities between HopZ3, HopZ1a and these Eop1 variants at the protein sequence level, led us to question if the latest modelling software could also reveal likely similarities between these effectors at the structural level.

### 2.2. AlphaFold2-Predicted Tertiary Structures of the “Erwinia-Pantoea” Eop1 Effectors Mimic the HopZ1a Structure, Providing Insight into Their Functional and Structural Characteristics

The tertiary structure of proteins and their 3D conformation are critical factors in determining protein function. The recently deciphered crystal structures of HopZ1a and PopP2 [20,36] have revealed structural and mechanistic details of the YopJ effectors. HopZ1a belongs to the “group III” clade of the YopJ family effectors, a clade populated by YopJ effectors from other *Pseudomonas* and *Erwinia* species [9]. Thus, HopZ1a is the nearest sequence homologue of Eop1 whose crystal structure has been deciphered [9,20]. We compared AlphaFold2 (AF2)-predicted tertiary structures of the six selected *Erwinia*-*Pantoea* clade Eop1 variants with the crystal structure of HopZ1a to derive clues about their structural and functional characteristics.

A tertiary structure superimposition analysis of the *Ea*246 Eop1 model with HopZ1a suggests that Eop1 variants adopt a HopZ1a-like conformation, with the respective three-dimensional positions of the catalytic and regulatory domains conserved; however, a HopZ1a-specific protein segment was discovered, which was absent in the tested Eop1 variants and replaced by a shorter loop (Supplementary Figure S4). Further investigation conducted via protein sequence alignment of HopZ1a with HopZ1b, HopZ1c and HopZ3 (outgroup) discovered a sudden drop in respective sequence alignment similarity and gaps at the site of this “HopZ1a specific region”, implying that the region may be undergoing active evolutionary selection and diversification, and thus may play an essential function in the YopJ clade of effectors. The same region was identified by Ma et al. [34] as being under the greatest positive selection. HopZ1a also has a much shorter disordered N-terminal region than HopZ3 and the Eop1 proteins. An analysis of disorder, potential molecular recognition features (MoRFs) and the secondary structural elements predicted by AlphaFold2 in this region suggests two likely MoRFs in this region (Supplementary Figure S5). A superimposition analysis between *Ea*246 and GCN5-related N-acetyltransferase (Supplementary Figure S6), another bacterial-origin acetyltransferase, suggests that no structural similarity exists between the YopJ acetyltransferase and other archetypal acetyltransferases. This indicates that these two acetyltransferases have probably evolved through independent pathways.

**Figure 2:**
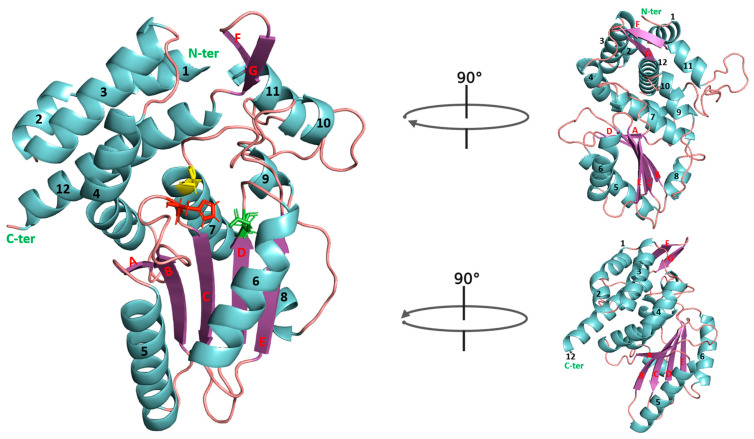
A detailed view of *the Erwinia amylovora* str. Ea246 Eop1 (AAF63400) tertiary structure predicted by AlphaFold2. The α-helices and β-sheets are colour coded with cyan and purple, respectively, and numbered (helices) or labeled with letters (β-sheets). The catalytic triad residues, i.e., histidine, glutamic acid and cysteine (H/E/C) are coloured red, green, and yellow, respectively.

**Figure 3:**
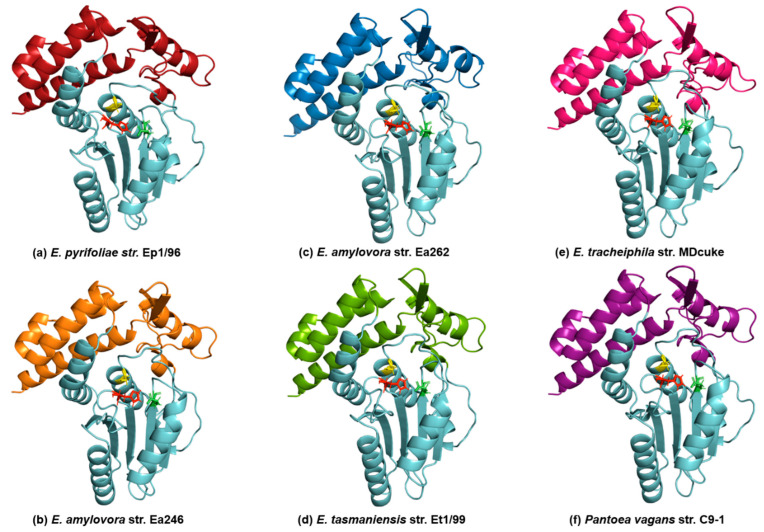
AlphaFold2 predicted tertiary structure models of the Eop1 variants of the *Erwinia-Pantoea* clade. The name of the resident species/strain of the corresponding structure is mentioned below the structures. The cyan-coloured structure represents the catalytic domain; the regulatory domains are presented in variable colours with each colour specific to one resident species. The catalytic triad residues, i.e., histidine, glutamic acid and cysteine (H/E/C), in the structures, are coloured red, green, and yellow, respectively.

AF2 models of the *E. amylovora* str. Ea246 Eop1 (employed as the archetypal Eop1 in the current study) and the five other chosen Eop1 variants are comprised of twelve α-helices and seven β-sheets (Figure 2 and Figure 3), assuming a HopZ1a-like structure. The crystal structure of HopZ1a has fourteen α-helices and eight β-sheets [20]. Similar to HopZ1a, the α-helices and β-sheets in our AF2 models form a closely packed complex with one catalytic domain sandwiched between two regulatory domains a.t each end of the protein sequence (Figure 2 and Supplementary Figure S2). The catalytic domain is comprised of five β-sheets and five α-helices, with β-sheets clustering between two α-helices from one side and three α-helices from the other side. Again, similar to HopZ1a, the deduced catalytic triad residues, i.e., histidine (H), glutamic acid (E) and cysteine, (C), are located at the centre of the probable catalytic domain in all *Erwinia*-*Pantoea* Eop1 variants. The histidine residue is located on a flexible loop bridging between the second and third β-sheets (“B” and “C” β-sheets in Figure 2 and in Supplementary Figure S3). In contrast, the glutamic acid and cysteine residues are found at the end of the fourth β-sheet (β-sheet “D”) and seventh α-helix, respectively (Figure 2). The structural modelling of all of the Eop1 variants was very similar and provided no obvious significant structural variation clue that might explain the inability of the E. tr_MDcuke allele to induce an HR in *N. tabacum*. Despite the overall structural similarity, there was significant primary sequence variation between the tested Eop1 variants (Supplementary Figures S1 and S2) that could result in a differential interaction with either a defence target or a resistance protein and account for this lack of HR.

**Figure 4:**
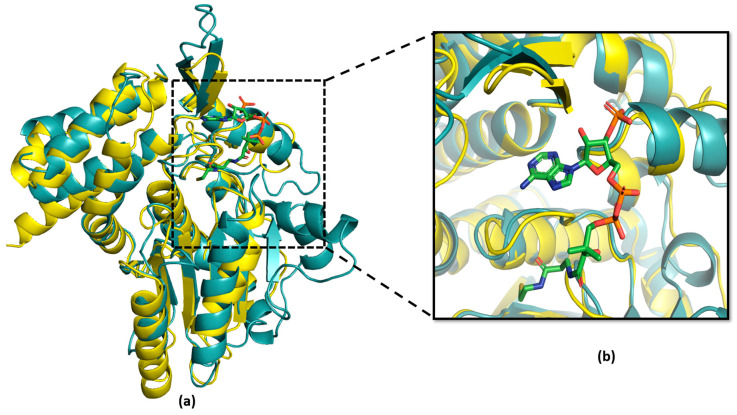
Analysis of acetyl coenzyme A (AcCoA) substrate-binding pocket in HopZ1a (cyan) and *Ea*246 Eop1 (yellow) via superimposition analysis: (**a**) Tertiary structure superimposition between HopZ1a and *Ea*246; (**b**) magnified view of the AcCoA-binding pocket forming α-helices in HopZ1a and *Ea*246 Eop1.

**Figure 5:**
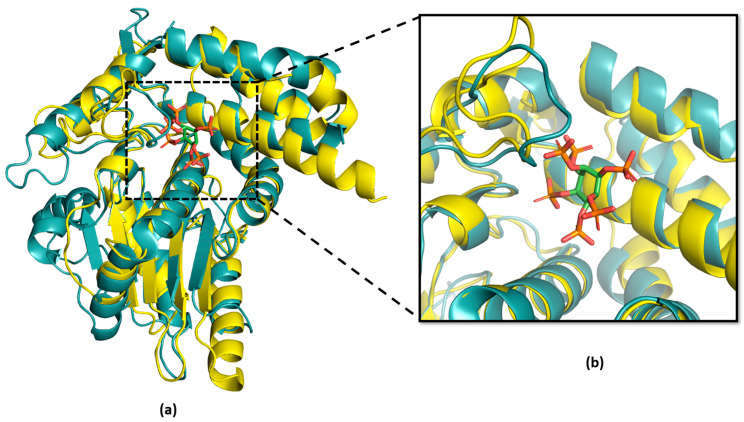
Analysis of Inositol hexakisphosphate (IP6) co-factor-binding pocket in HopZ1a (cyan) and *Ea*246 (yellow): (**a**) Tertiary structure superimposition of HopZ1a and *Ea*246 Eop1; (**b**) magnified view of the “IP6-binding pocket” forming α-helices in HopZ1a and *Ea*246 Eop1.

The superimposition analysis also discovered potential substrate-binding and co-factor-binding pockets in the Eop1 variants; this mimicked the AcCoA and IP6 binding pockets in HopZ1a, respectively. To further analyse this observation, the HopZ1a tertiary structure bound to IP6 and AcCoA (PDB: 5KLQ) was superimposed onto the Ea246 Eop1 structure (Figure 4 and Figure 5). As anticipated, the IP6 and AcCoA-binding pockets in HopZ1a aligned closely with the predicted secondary structure of the putative binding pockets of the analysed Eop1 variants. Moreover, the amino acid residues that interact with IP6 and AcCoA in HopZ1a—directly or via water molecule-mediated hydrogen bonds—were also found to be substantially conserved in all the tested Eop1 variants (Supplementary Figure S7). Collectively, these results strongly suggest that, similar to HopZ1a, the Eop1 variants probably require the eukaryote-specific co-factor IP6 for their activation. We deduce that IP6-mediated activation would likely result in the effector’s conformation change, allowing AcCoA binding, which ultimately facilitates the acetylation process. Overall, these structural and sequence conservation analyses strongly imply that Eop1 effectors, like their structural homologues HopZ1a and PopP2, are acetyltransferases. This finding also implies the probable conservation of function of the catalytic residues in Eop1 variants. Therefore, we set out to test the functional importance of each of these putative catalytic residues by employing targeted mutational analyses.

### 2.3. Erwinia amylovora Eop1 Effector (Ea246) Utilises a Catalytic Dyad with a Conserved Histidine Residue Required for Catalysis

No naturally occurring amino acids are capable of functioning as strong nucleophiles in catalysis by themselves. Consequently, many enzymes have evolved with a unique feature in which an amino acid trio works in unison to generate a strong nucleophile capable of inducing catalysis. Within the YopJ superfamily of evolutionarily conserved bacterial effectors, the catalytic trio is usually considered to consist of H, E and C, in which the residues act as a base, acid and nucleophile, respectively [9,37]. Previous analyses have suggested that all catalytic residues are of functional importance to the YopJ effectors, which would explain why they are largely conserved amongst their sequence homologues [25]. To analyse the catalytic triad conservation in the Eop1-like branch of YopJ variants, we performed a BLASTp search using the Ea246 Eop1 variant protein sequence as a “query sequence”. Despite a considerable difference in the protein sequence identity of the retrieved sequences (ranging from 30% to 100%), the catalytic triad residues were found to be conserved in virtually all the Eop1-like sequences; moreover, they were perfectly conserved in a subset with 75% or greater identity (Supplementary Figure S8).

The broad conservation of the catalytic triad residues in the YopJ variants, as reported in the previous literature, combined with the strong resemblance of their 3D conformation with the HopZ1a catalytic triad conformation, led us to postulate that the catalytic triad residues could play a crucial role in the Ea246 Eop1-induced catalysis in *N. tabacum*. We assessed the functional importance of the putative triad residues indirectly via the HR-eliciting activity of Eop1 in *N. tabacum.* To test the hypothesis, site-directed mutagenesis was used to create catalytic triad mutants, which were then tested by using the HR assay in *N. tabacum*.

**Figure 6:**
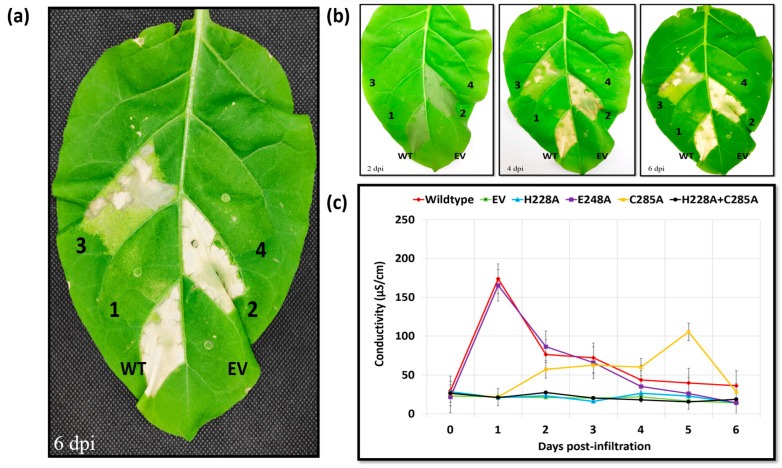
Transient expression analysis of Ea246 Eop1 catalytic triad mutants: (**a**) Hypersensitive response-induced cell death phenotype induced by the catalytic triad mutants; (**b**) analysis of the progression of HR-induced cell death elicited by the catalytic triad mutants over 6 days; (**c**) electrolyte leakage assay data from the leaf discs infiltrated with *Agrobacterium* cells harbouring the expression clones of the catalytic triad mutants. The annotations are as follows: WT, wild type; EV, empty vector; 1, H228A; 2, E248A; 3, C285A; 4, H228A + C285A (Ea246 Eop1 double mutant). The experiments were repeated 5 times with similar results. The images were taken on the days indicated on the bottom left of the leaf images. The leaf width at 6 dpi was approximately 7 cm.

As observed in the above experiments, the transient expression of the wild-type Eop1 protein Ea246 again triggered a strong HR at 24 hpi (Figure 6a). Surprisingly, the glutamic acid-substituted mutant (E248A) also produced a similarly strong HR. In contrast, no HR-induced cell death was observed upon agroinfiltration of the histidine mutant (H228A) of Eop1. Intriguingly, the transient expression of the cysteine mutant (C285A) elicited a much slower HR that developed over 6 days. Cell death was first observed in small patches at 2 dpi and gradually grew to encompass the infiltrated region by the sixth day (Figure 6b). To further validate the activities of the cysteine and histidine mutants, a double mutant was created by combining the C285A and H228A mutations in the Ea246 Eop1 backbone. The double mutant did not trigger any HR (Figure 6a), presumably because of the epistatic nature of the mutation of the histidine residue in Ea246 Eop1 when combined with the cysteine mutation.

Electrolyte leakage assays were also performed to quantify and validate the HR assay result of the Ea246 catalytic triad mutants (Figure 6c). As expected, the electrolyte leakage data corroborated phenotypic observations with the HR assay. At 24 hpi, leaf discs infiltrated with *Agrobacterium* carrying clones expressing the wild type and the glutamic acid mutant exhibited rapid and significant ion leakage, with conductivity exceeding 150 μS/cm. In contrast to the wild type, the histidine and double mutants exhibited significantly less ion leakage upon infiltration, even after prolonged incubation. However, progressive ion leakage, indicated by a gradual increase in conductivity, was detected in leaf discs infiltrated with *Agrobacterium* harbouring the cysteine mutant. With this mutant, the electrolyte leakage-induced conductivity was observed to rise after 1 dpi, gradually increased until the fourth day and culminated with a peak value of 100 μS/cm on the subsequent day. The ion leakage trend for the cysteine mutant provided further proof that the observed ion leakage coincided with progressive HR-like cell death. 

The conservation analysis, conducted in this study, indicates that the catalytic triad residues, C/H/E, are evolutionarily conserved in the YopJ effectors, implying potential functional significance in the YopJ effectors. Moreover, the 3D structural conformation of the triad residues, as also seen in the crystal structures of HopZ1a and PopP2 [20,35], strongly suggested that these catalytic residues would be the central drivers of the enzymatic activity in different YopJ variants. For the first time, we attempted to test and validate the involvement of all the putative catalytic residues in the enzymatic activity in the YopJ effectors via mutation analysis. The analysis produced a set of novel findings that presents a new picture of the putative catalytic triad and the residues “actually” involved in catalysis. Firstly, unlike the wild type or the cysteine mutant, the histidine mutant failed to trigger any HR-induced cell death in the non-host plant *N. tabacum*, indicating that the effector is likely to function via an enzymatic mechanism. Secondly, the lack of involvement of the conserved glutamic acid, validated by electrolyte leakage assay, suggests that the tested *E. amylovora* Eop1 effector functions as a “dyad” rather than a “triad”, involving only the acid-nucleophile duo. Finally, the phenomenon of a slow progressive HR induced by the Ea246 Eop1 catalytic cysteine mutant suggests two hypotheses:

(a) One hypothesis predicts that C285 is not the “actual” nucleophile, but another amino acid residue functioning as the nucleophile in the catalysis is proximal to the mutated C285 residue. The C285A mutation could induce a conformation deformity in the catalytic pocket which interferes with, and impedes the catalytic activity, thus resulting in the slow catalysis, and consequently slower HR. However, AF2-predicted structures of the catalytic triad residue-mutated variants of Ea246 Eop1 exhibited no significant deviation from the wild-type structure (Supplementary Figure S9). Therefore, this possibility was deduced to be unlikely.
(b) An alternate hypothesis predicts the presence of a “secondary nucleophile” that compensates for the loss of the “primary nucleophile” in Ea246 Eop1. Simply put, the proposal is that, whenever present, the proposed nucleophile, C285, functions as the “primary” nucleophile; however, in its absence (as is the case for the C285A mutant), another nearby “secondary nucleophile” with potentially weaker nucleophilic activity can function as a “substitute nucleophile” and still drive a slower rate of catalysis in this Eop1 variant.

Considering the above hypotheses, the involvement of another residue functioning as a nucleophile apart from C285 in Ea246 Eop1 seemed likely. Therefore, we set out to test these hypotheses.

### 2.4. Ea246 Eop1 Retains Its Ability to Function via a Substitute Nucleophile in the Absence of the Primary Nucleophile

The results from the catalytic triad mutation experiment presented in the previous section suggested that a “secondary nucleophile” may compensate for the loss of the primary nucleophile, C285, in the Ea246 Eop1 variant. To identify the residue that could potentially function as a secondary nucleophile, first, we looked for amino acids that were structurally and chemically similar to cysteine and also function as nucleophiles in other catalytic mechanisms.

The amino acid, serine, is structurally very similar to cysteine, differing only at the beta-carbon site in the R-group. Cysteine possesses a thiol group (–SH) on its beta carbon, whereas serine has a hydroxyl group (–OH). Furthermore, the replacement of the oxygen atom with sulphur in cysteine results in only minor differences in bond angles and bond length, suggesting that serine is an isosteric and possibly isostructural double of cysteine [38]. Chemically, cysteine and serine exhibit similar properties; however, they differ significantly in their side-chain functional group’s “acid dissociation constant” (pKa) values, which are 15.9 and 9.5 for the hydroxyl group [39,40] and thiol group [40,41,42] in serine and in cysteine, respectively. Despite this difference in the pKa values, serine proteases with serine residue functioning as nucleophiles in the catalytic triad are abundant naturally [37,43,44]. This similarity to cysteine protease function suggests that a serine residue is most likely to be the secondary nucleophile in Ea246 Eop1.

**Figure 7:**
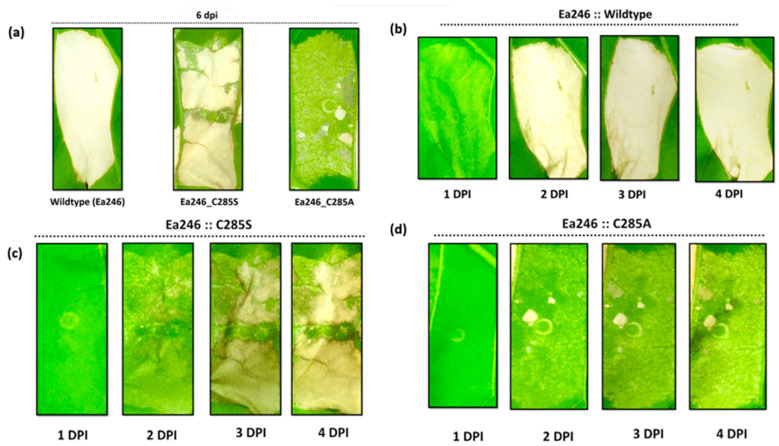
Analysis of the hypersensitive response elicited by alanine and serine mutants of the thiol Eop1 in *Nicotiana tabacum*: (**a**) A comparison of the wild type and the alanine and serine mutants of Ea246 at 6 days post-infiltration (dpi); analysis of the progressive HR over 4 days in (**b**) wild type, (**c**) serine mutant of the Ea246_C285, and (**d**) alanine mutant of Ea246_C285.

**Figure 8:**
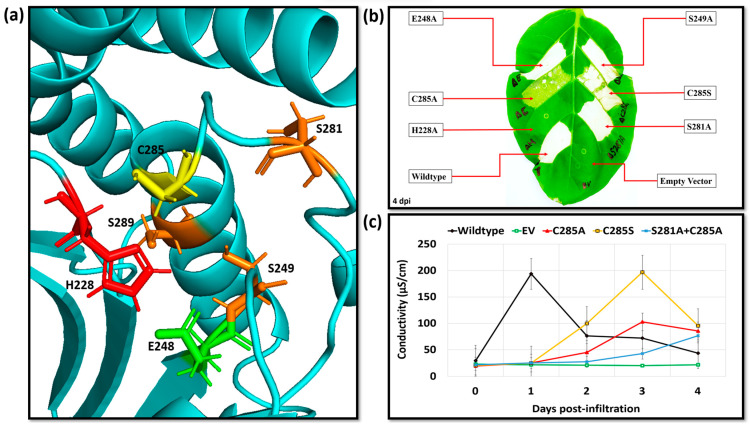
Identification of the putative secondary nucleophiles via proximity analysis to the predicted catalytic triad and functionally important histidine residue: (**a**) Proximity analysis, the *Ea*246 catalytic triad residues’ colour codes are red (histidine (H228)), green (glutamic acid (E248)), yellow (cysteine (C285)), serine residues identified as “putative secondary nucleophiles” (S249, S281 and S289) are presented via orange colour, and the Ea246 protein backbone is represented by the cyan colour; (**b**) transient expression analysis of the Ea246 Eop1 predicted catalytic triad mutants (H228A/E248A/C285A), putative secondary nucleophiles (S281A and S249A) and hydroxyl (serine) mutant of the thiol nucleophile (C285S). (**c**) electrolyte leakage assay data from leaf discs infiltrated with Agrobacterium expressing Eop1 clones with alanine and serine mutants of the thiol Eop1 (Ea246).

Given the reasoning above, we sought proof that a serine residue in Ea246 Eop1 variant might be able to function as a nucleophile. A serine mutant of the putative cysteine nucleophile effector was created and analysed to partially test this hypothesis. When transiently expressed via *Agrobacterium*, the C285S mutant of Ea246 Eop1 elicited HR in tobacco that began phenotypically as multiple specks at 2 dpi and progressed more quickly than C285A-induced HR, but less quickly than that in the wild type (Figure 7). The HR encompassed nearly all the infiltrated region by the end of the fourth day. These results were validated via ion leakage assays, which revealed a conductivity trend paralleling the visual results observed from HR assay (Figure 8b,c). Together, these results support the hypothesis that a serine residue can function as a nucleophile in Ea246 Eop1.

The second step in solving the “secondary nucleophile” puzzle was to find the actual serine residue(s) that could function as the substitute nucleophile in the 393-residue-long protein sequence. To tackle this problem, we devised a structure-informed approach to search for and to identify the serine residues sharing high 3D proximity to the (now experimentally validated and functionally important) histidine residue, H228.

The rationale for this approach was that the nucleophilic activity is critical for the YopJ effectors-driven catalysis; moreover, during catalysis, the catalytic histidine functions as a proton acceptor, assisting in the deprotonation of the residue serving as a nucleophile. The results presented in the previous section also indicate that the histidine residue is crucial for catalysis. Consequently, it was hypothesised that a histidine-neighbouring serine residue would be most likely to function as a secondary nucleophile. The AF2-produced tertiary structure model of Ea246 Eop1 was used to identify the serine residues likely to share high proximity to the histidine residue (within 10 Å, three dimensionally). The proximity analysis identified three residues: S249, S281 and S289, with proximities of 5.9 Å, 8.7 Å and 6.1 Å, respectively, to the likely proton-accepting atom of histidine (Figure 8a). Two of these residues, S249 and S281, were widely conserved (Supplementary Figure S8) and present on more flexible loop regions within the active site, whereas the third residue was in a more-or-less fixed position on the same helix as the primary cysteine nucleophile. This third residue was also poorly conserved between Eop1 variants (Supplementary Figure S8). In fact, apart from HopZ3_Psy_, serine 289 was present only in two of the six Eop1 variants we tested. We reasoned that the lack of conservation of S289 and the flexibility of the other two serine residues increased the likelihood that S249 or S281 could compensate for the loss of the cysteine nucleophile, while retaining the overall shape of the catalytic pocket.

First, we focused on confirming that the identified serine residues, S249 and S281, do not function as primary nucleophiles in the presence of the predicted primary nucleophile, C285, by mutating them to alanine (Figure 8b,c). The results obtained from this analysis confirmed that mutation of residues S249 and S281 did not affect Ea246 ability to induce an HR. This observation also suggests that it is likely C285 is, in fact, the primary nucleophile. In line with the second step in the approach, we created and tested a double mutant of the residue in the most flexible part of the pocket: S281A and C285A in the Ea246 Eop1 backbone. This resulted in gradually progressing HR, a phenotypically similar effect to the single mutant C285A-induced HR (Figure 8c). Thus, we concluded that S281 is probably also not acting as a substitute secondary nucleophile. Our results do not rule out the possibility that one of the other two potential nucleophiles (although less likely) is actually responsible for the secondary nucleophilic activity and inducing HR.

### 2.5. Negative Charge at the Catalytic Pocket Provides a Suitable Environment for the Serine Residue to Function as a Secondary Nucleophile in Ea246 Eop1

Given the significant reduction in ability of Eop1 Ea246 to induce HR upon its mutation to serine or alanine, it is very likely that cysteine 285 functions as the primary nucleophile. However, one result does call into question the suggestion that this residue is the nucleophile, namely, how can an enzyme that is supposed to function with a nucleophile still show activity (albeit significantly slower) when its primary nucleophile is mutated to a residue that is incapable of supplying nucleophilic activity (alanine)? Thus, we set out to obtain additional data that could help explain the secondary nucleophile hypothesis. In the previous section, we showed that the serine mutant of the cysteine nucleophile (C285S) still elicits HR-like cell death when transiently expressed in *N. tabacum*, supporting the idea that another histidine-neighbouring serine residue might also be able act as a secondary nucleophile in Ea246 Eop1.

There is a growing realisation that the electrostatic environment of enzymes, particularly near the catalytic motifs, affect catalysis [40,45]. Cysteine and serine peptidase, which have been assumed to function via a nucleophile, acid and base catalytic triad, catalyse via different mechanisms. Cysteine peptidases catalyse, in a stepwise manner, via the ion pair intermediate form, involving the formation of a thiolate anion functioning as a nucleophile [46]. In contrast, serine peptidases catalyse in a “concerted” manner, with the serine nucleophile formation and attack occurring simultaneously [47]. Interestingly, in cysteine peptidases, the thiolate anion is typically stabilised via hydrogen bonds and positive electrostatic potential [46,48,49], whereas, in serine peptidases, the negative electrostatic potential was observed to be crucial for transition state stabilisation during catalysis [44,50]. Furthermore, Gisdon et al. [40] pointed out that the negative electrostatic environment in serine peptidase was crucial to destabilise the serine anion, which aids in increasing its nucleophilicity towards its corresponding substrate(s).

**Figure 9:**
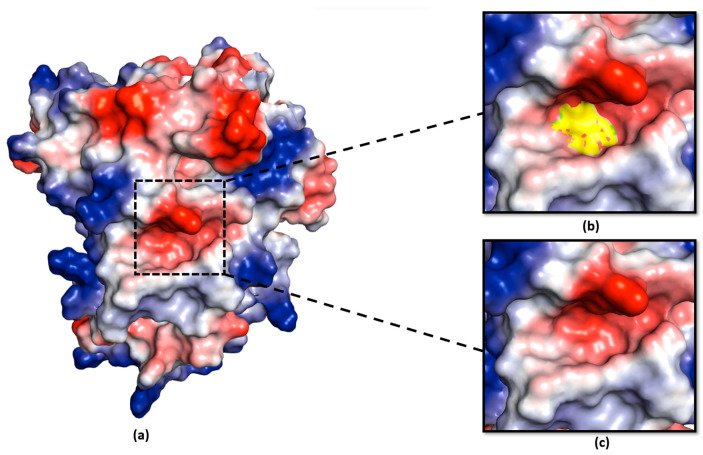
Ea246 Eop1 catalytic motif electrostatic potential analysis: (**a**) PyMOL-generated vacuum electrostatic potential model of Ea246; (**b**) Ea246 catalytic triad-contributed surface in the catalytic motif (yellow); (**c**) electrostatic potential at the site of the catalytic triad-contributed surface in Ea246.

**Figure 10:**
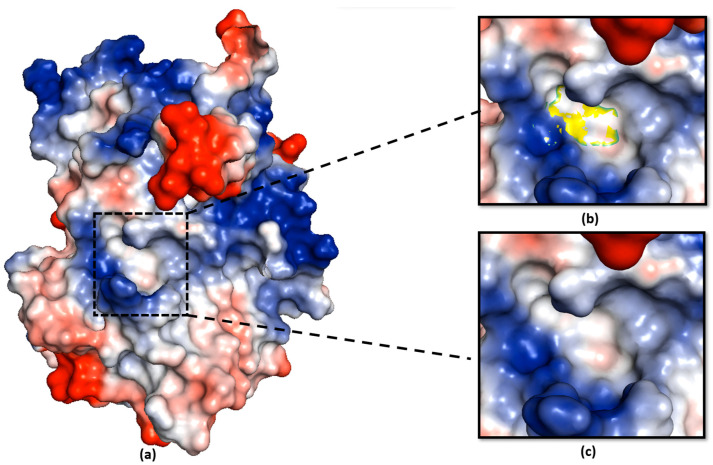
HopZ1a catalytic motif electrostatic potential analysis: (**a**) PyMOL-generated vacuum electrostatic potential model of HopZ1a; (**b**) HopZ1a catalytic triad-contributed surface in the catalytic motif (yellow); (**c**) electrostatic potential at the site of the catalytic triad-contributed surface in HopZ1a.

We analysed the electrostatic potential environment of the Ea246 Eop1, its catalytic triad mutants and HopZ1a, to determine the potential contribution of the electrostatic environment in catalysis. Intriguingly, on the one hand, careful examination of the electrostatic nature of the predicted models revealed that the Ea246 Eop1 catalytic motif region was electrostatically negative (Figure 9), providing a favourable environment for a secondary serine in proximity to the active site histidine to act in nucleophile-driven catalysis. HopZ1a, on the other hand, was discovered to have an electrostatically neutral environment (Figure 10), which could explain why its cysteine mutant did not trigger any HR in *Arabidopsis* [30] despite having some of the potential secondary nucleophile residues conserved (Supplementary Figures S7 and S8). Alternatively, this supports the possibility that S249 (substituted by a P in HopZ1a) is actually the secondary nucleophile in Ea246 Eop1.

Compared with the wild type, the slower rate of the HR phenotype observed upon the transient expression of C285S (as shown in Figure 7) can be attributed to the difference between the thiol and hydroxyl nucleophile pKa values in cysteine and serine, respectively, or Ea246 YopJ catalysis via a “dyad” or both. This affects the catalysis rate because the lower pKa value of cysteine makes it a better nucleophile as its deprotonation is comparatively easier than that of serine. 

The gradual HR phenotype observed upon the transient expression of C285A could be explained by a serine residue acting as a secondary nucleophile in the absence of the primary cysteine nucleophile. The gradual nature of the HR could be a result of the distance between the putative secondary nucleophile and the histidine residue, resulting in slow catalysis. Here, the electrostatically negative environment at the Ea246 Eop1 active site provides a suitable environment for serine substitution or the neighbouring-serine residues to function as primary and secondary nucleophiles, respectively, in a catalytic dyad system.

## 3. Materials and Methods

### 3.1. Bacterial Strains and Protocols

*Escherichia coli* (TOP10) and *Agrobacterium tumefaciens* (GV3101) cells were cultured on the Luria–Bertani (LB) solid medium at 37 °C and 28 °C, respectively, for 28–36 h with appropriate antibiotics.

### 3.2. Plant Material

*Nicotiana tabacum* “Samsun” plants used in this study were grown in the controlled environment of a glass house maintained at 22 °C with long-day conditions of 16:8 h of light and darkness, under optimal humidity.

### 3.3. Agrobacterium tumefaciens Mediated Transient Expression Assays

*Agrobacterium tumefaciens* (GV3101) were grown overnight in an LB liquid medium containing appropriate selective antibiotics at 28 °C and 200 rpm. The overnight culture was centrifuged, and the bacterial cell pellet was resuspended in an infiltration buffer (10 mM MES (pH 7.2) and 10 mM MgCl_2_). The bacterial concentration (OD_600_) of the infiltration solution was adjusted to the desired OD_600_ before infiltration in the *N. tabacum* plants via 1 mL needleless syringe. Note: All transient expression assay results were reported after replicating the results at least three or more times.

### 3.4. Electrolyte Leakage Assays

For the electrolyte leakage assay, 21–25 day-old *N. tabacum* “Samsun” leaves were infiltrated with *A. tumefaciens* (GV3101)-harbouring expression clones, carrying the gene of interest. Following that, two leaf discs of 10 mm diameter were collected from the similar aged leaves from separate plants on the days indicated on the graph plots. Next, the leaf discs were gently washed in 25 mL Milli-Q^®^ (MQ) water by shaking for 10 min. Then, the leaf discs were strained, rinsed, and 2 leaf discs were gently placed in 2 mL MQ water to collect electrolyte leakage from the plant cells, and shaken for 2 h at 150 rpm. Next, the conductivity was measured by pipetting 80 μL of MQ-water to measure the ion leakage using a conductometer (Horiba Scientific, Stanmore, UK). The graph for each tested construct, on the indicated days, was plotted based on the mean value from four replicates (*n* = 4).

### 3.5. Bioinformatic Methods

For multiple sequence alignment, all the polypeptide sequences were aligned via Clustal Omega [51] using default settings in Geneious Prime, version 2022.0.1 (https://www.geneious.com/).

### 3.6. Protein Tertiary Structure Models

The tertiary structure models of the Eop1 variants tested in the current study were produced via AlphaFold2 Collab v2.3.2 [52] open source code (https://colab.research.google.com/github/deepmind/alphafold/blob/main/notebooks/AlphaFold.ipynb, from 1 March 2022–31 July 2022) and visualised via PyMOL, version 2.0 (Schrödinger, LLC, Portland, OR, USA), a Molecular Graphics System. The regulatory and catalytic domains within the YopJ variants, along with the catalytic triad residues site, were identified using the protein sequence alignment with HopZ1a. The sequence length of the domains is listed in Supplementary Table S2, with the catalytic residues site listed in Supplementary Table S3. The “vacuum electrostatic model” of the tested YopJ variants was generated via PyMOL. Superimposition analysis of the AF2 models and the crystal structure was also carried out in the PyMOL V2.0 software.

### 3.7. Site-Directed Mutagenesis

The Ea246 Eop1 effector mutants were generated via site-directed mutagenesis, as guided by the QuikChange™ system with slight modifications, i.e., using two steps PCR along with Q5^®^ High-Fidelity DNA Polymerase (NEB, M0491S) enzyme. The sequence of the primer pairs used to generate the mutants are listed in Supplementary Table S4.

## 4. Conclusions

### Novel Findings from the Analysis of the Eop1 Branch of YopJ Effectors

We took a novel multi-pronged approach to analyse the Eop1 effector group by taking advantage of accurate structural modelling enabled by AlphaFold2. The results and modelling imply several interesting novel findings that, in turn, provide insights into the likely function, evolution, and adaptation that Eop1 effector alleles undergo as they battle with plants’ multiple surveillance systems. This study also highlights some potential inadequacies in single residue knock-out strategies (often used to claim that a protein acts via a catalytic triad), even when the catalytic triad is well conserved across a broad set of orthologues (as is the case for the Eop1 clade that we analysed).

We found that most of the Eop1 variants we tested could, like HopZ3, induce an HR in *N. tabacum*. HopZ3 is a particularly interesting effector, as it was one of the first effectors known to be able to suppress HRs induced by a number of other *P. syringae* effectors [31]. A potential mechanism explaining HopZ3 suppression abilities (acetylating several plant and bacterial members of the RPM1 resistance gene complex) was identified by Lee et al. [18]. Abilities to suppress multiple R-proteins that trigger resistance make these types of effectors powerful tools within the pathogens armoury of weapons to escape or blunt the recognition capabilities of host plants. Since our study shows that Eop1 effectors share IP6 and acetyl CoA-binding domains and are structurally largely conserved with respect to HopZ1a (and other YopJ proteins); therefore, they are likely to perform similar acetylating functions. We deduce it is also likely that they may similarly suppress the resistance-triggering “legacy” of other *Erwinia* effectors. In this respect, it was particularly interesting that the Eop1 variant we discovered, which was unable to trigger an HR in *N. tabacum* (*E. tr*_Mdcuke), has also been shown to increase the virulence of squash strains on muskmelon [33]. Similar phenomena have been found with other effector-resistance pairs in other plant–pathogen interactions. Therefore, the *E. tr*_Mdcuke Eop1 variant effector may be free to suppress other resistance triggering mechanisms in muskmelon without an accompanying HR or compromising *Erwinia* pathogenicity. Understanding such interplays of effectors is vital for us to be able to design future durable resistance strategies against these pathogens. We note that the Eop1 clade of effectors occurs in important pathogens of multiple Rosaceae crops such as apple and pear, and that these pathogens are responsible for renewed outbreaks of diseases caused by *Erwinia* species, such as the recent fire blight outbreak on pear in the Republic of Korea [53].

The approach also revealed other surprising novel findings. These include the discovery that there are two variable domains that differ between the YopJ proteins (from our comparative AF2 modelling). YopJ effectors have already been shown to target a long list of different hosts’ defence and pathogen effector proteins that include RIN4, RIPK, WRKY transcription factors, MAP kinases, NF-ƙB, JAZ, ACIP, tubulin, AvrB3 and AvrRpm1_Psy_ [9,18,20,30,36,54,55]. The HopZ1a specific region, which we deduce may be involved with binding targets, is located within the catalytic domain (just adjacent to the regulatory domain). It is on the surface and on one side of the effector, meaning its position is ideal for it to play a role in facilitating the binding of different protein targets in different YopJ effectors. The new group of effectors that we analysed (HopZ3 and six Eop1 proteins) all have a much longer N-terminal region than HopZ1a; whilst this region is highly disordered, it contains a number of predicted MoRFs. One of these MoRFs at the extreme N-terminus is a common location for N-myristoylation-directed membrane-anchoring motifs, whereas a second predicted MoRF coincides with predicted alpha helices in the Eop1 Ea246 and HopZ3 AlphaFold2 models and coincides with a large dip in the disorder predictor VX-LT (Supplementary Figures S5 and S7). This region is conserved between this group of YopJ proteins, and therefore is also a potential protein–protein interaction motif involved in host target protein binding.

Our finding that there may be a serine capable of acting as a substitute nucleophile provides new insight into the likely function and evolution of catalytic triads in effectors. Although our structural insights allowed us to test some combinations of mutations of possible primary and secondary nucleophiles, we were unable to pinpoint an unambiguous explanation for these results; more mutation combinations remain to be tested in future. In hindsight, focusing on the conserved possible secondary nucleophiles, combined with our choice of Eop1_Ea246 as the effector to modify, may have been unfortunate. It was one of only two of the effectors used in our study, with a third alternate secondary nucleophile serine that we did not test as it was poorly conserved. It is possible that if we had tested the mutations in the background of one of the other four Eop1 alleles (which do not have this serine), the cysteine to alanine mutation might have completely knocked out the catalytic function. Given the high degree of conservation between the catalytic pocket and the lack of disturbance of the AF2 models created by the mutations, we still favour the idea that one of the serines, fortuitously, can act as an inefficient secondary nucleophile. Our current thinking is that this property could, in fact, be specific to the Eop1_Ea246 allele and others carrying a serine in an equivalent position to S289. In terms of proximity, this serine is just one full turn away and on the same alpha helix that carries the cysteine primary nucleophile, and 6.1 Å away from the histidine atom requiring nucleophilic attack. This fits well with the reduced catalytic efficiency that we deduce from the slower HR reaction of the cysteine mutant.

Conservation of a putative catalytic triad, combined with the loss of enzyme activity following mutation of just one member of the putative triad, is often used to suggest proof of function of the entire catalytic triad. Considering our results, this is probably insufficient proof. At the very least, we would suggest that mutations in all three putative catalytic triad residues should be tested to see if the enzyme is likely to perform as a triad or dyad. Modelling the structure of such enzymes using new capabilities such as AlphaFold2 should also be used to assess the positions of catalytic residues and to help to explain results, particularly if they do not completely match the expected loss of activity when these residues are mutated (as we found). In the light of other theories about the importance of electrostatic charge in the catalytic capability of enzyme active sites, our results force us to rethink the potential for enzymatic mechanisms to show subtle but important differences between structurally related proteins. An understanding of how genetic drift associated with localized charge potential might be able to play a role in adapting enzymatic function of effectors in response to the dynamic interaction between pathogens and their key host defence targets, would provide a new framework for exploring ways that the pathogen effector armoury can respond to the pressures of plant defence.

## Data Availability

Extra data is available in the Supplementary File or upon request from the first author or corresponding author.

## Supplementary Materials

The following supporting information can be downloaded at: https://www.mdpi.com/article/10.3390/ijms241914664/s1. References [56,57,58,59,60,61,62,63,64] are cited in the supplementary materials.

## References

[1] Jones J.D., Dangl J.L. (2006). The plant immune system. Nature.

[2] Boller T., Felix G. (2009). A renaissance of elicitors: Perception of microbe-associated molecular patterns and danger signals by pattern-recognition receptors. Annu. Rev. Plant Biol..

[3] Afzal A.J., da Cunha L., Mackey D. (2011). Separable fragments and membrane tethering of Arabidopsis RIN4 regulate its suppression of PAMP-triggered immunity. Plant Cell.

[4] Rathore J.S., Ghosh C., Singh A., Singh I.K. (2018). Pathogen-Associated Molecular Patterns and Their Perception in Plants. Molecular Aspects of Plant-Pathogen Interaction.

[5] Głowacki S., Macioszek V.K., Kononowicz A.K. (2011). R proteins as fundamentals of plant innate immunity. Cell Mol. Biol. Lett..

[6] Kourelis J., van der Hoorn R.A.L. (2018). Defended to the Nines: 25 Years of Resistance Gene Cloning Identifies Nine Mechanisms for R Protein Function. Plant Cell.

[7] Yang S., Li J., Zhang X., Zhang Q., Huang J., Chen J.Q., Hartl D.L., Tian D. (2013). Rapidly evolving R genes in diverse grass species confer resistance to rice blast disease. Proc. Natl. Acad. Sci. USA.

[8] Lewis J.D., Lee A., Ma W., Zhou H., Guttman D.S., Desveaux D. (2011). The YopJ superfamily in plant-associated bacteria. Mol. Plant Pathol..

[9] Ma K.W., Ma W. (2016). YopJ Family Effectors Promote Bacterial Infection through a Unique Acetyltransferase Activity. Microbiol. Mol. Biol. Rev..

[10] Orth K., Xu Z., Mudgett M.B., Bao Z.Q., Palmer L.E., Bliska J.B., Mangel W.F., Staskawicz B., Dixon J.E. (2000). Disruption of signaling by Yersinia effector YopJ, a ubiquitin-like protein protease. Science.

[11] Le Roux C., Huet G., Jauneau A., Camborde L., Trémousaygue D., Kraut A., Zhou B., Levaillant M., Adachi H., Yoshioka H. (2015). A receptor pair with an integrated decoy converts pathogen disabling of transcription factors to immunity. Cell.

[12] Sarris P.F., Duxbury Z., Huh S.U., Ma Y., Segonzac C., Sklenar J., Derbyshire P., Cevik V., Rallapalli G., Saucet S.B. (2015). A Plant Immune Receptor Detects Pathogen Effectors that Target WRKY Transcription Factors. Cell.

[13] Cheong M.S., Kirik A., Kim J.G., Frame K., Kirik V., Mudgett M.B. (2014). AvrBsT acetylates Arabidopsis ACIP1, a protein that associates with microtubules and is required for immunity. PLoS Pathog..

[14] Jiang S., Yao J., Ma K.W., Zhou H., Song J., He S.Y., Ma W. (2013). Bacterial effector activates jasmonate signaling by directly targeting JAZ transcriptional repressors. PLoS Pathog..

[15] Lee A.H., Hurley B., Felsensteiner C., Yea C., Ckurshumova W., Bartetzko V., Wang P.W., Quach V., Lewis J.D., Liu Y.C. (2012). A bacterial acetyltransferase destroys plant microtubule networks and blocks secretion. PLoS Pathog..

[16] Lewis J.D., Wu R., Guttman D.S., Desveaux D. (2010). Allele-specific virulence attenuation of the *Pseudomonas syringae* HopZ1a type III effector via the Arabidopsis ZAR1 resistance protein. PLoS Genet..

[17] Lewis J.D., Lee A.H., Hassan J.A., Wan J., Hurley B., Jhingree J.R., Wang P.W., Lo T., Youn J.Y., Guttman D.S. (2013). The Arabidopsis ZED1 pseudokinase is required for ZAR1-mediated immunity induced by the *Pseudomonas syringae* type III effector HopZ1a. Proc. Natl. Acad. Sci. USA.

[18] Lee J., Manning A.J., Wolfgeher D., Jelenska J., Cavanaugh K.A., Xu H., Fernandez S.M., Michelmore R.W., Kron S.J., Greenberg J.T. (2015). Acetylation of an NB-LRR Plant Immune-Effector Complex Suppresses Immunity. Cell Rep..

[19] Mittal R., Peak-Chew S.Y., Sade R.S., Vallis Y., McMahon H.T. (2010). The acetyltransferase activity of the bacterial toxin YopJ of Yersinia is activated by eukaryotic host cell inositol hexakisphosphate. J. Biol. Chem..

[20] Zhang Z.M., Ma K.W., Yuan S., Luo Y., Jiang S., Hawara E., Pan S., Ma W., Song J. (2016). Structure of a pathogen effector reveals the enzymatic mechanism of a novel acetyltransferase family. Nat. Struct. Mol. Biol..

[21] Mukherjee S., Keitany G., Li Y., Wang Y., Ball H.L., Goldsmith E.J., Orth K. (2006). Yersinia YopJ acetylates and inhibits kinase activation by blocking phosphorylation. Science.

[22] Trosky J.E., Li Y., Mukherjee S., Keitany G., Ball H., Orth K. (2007). VopA inhibits ATP binding by acetylating the catalytic loop of MAPK kinases. J. Biol. Chem..

[23] Adeolu M., Alnajar S., Naushad S., Gupta R.S. (2016). Genome-based phylogeny and taxonomy of the ‘*Enterobacteriales*’: Proposal for *Enterobacterales* ord. nov. divided into the families *Enterobacteriaceae*, *Erwiniaceae* fam. nov., *Pectobacteriaceae* fam. nov., *Yersiniaceae* fam. nov., *Hafniaceae* fam. nov., *Morganellaceae* fam. nov., and *Budviciaceae* fam. nov. Int. J. Syst. Evol. Microbiol..

[24] Janda J.M., Abbott S.L. (2021). The Changing Face of the Family Enterobacteriaceae (Order: “*Enterobacterales*”): New Members, Taxonomic Issues, Geographic Expansion, and New Diseases and Disease Syndromes. Clin. Microbiol. Rev..

[25] Yuan X., Hulin M.T., Sundin G.W. (2021). Effectors, chaperones, and harpins of the Type III secretion system in the fire blight pathogen *Erwinia amylovora*: A review. J. Plant Pathol..

[26] Zhao Y., Gross D.C., Lichens-Park A., Kole C. (2014). Genomics of Erwinia amylovora and Related Erwinia Species Associated with Pome Fruit Trees. Genomics of Plant-Associated Bacteria.

[27] Panstruga R., Moscou M.J. (2020). What is the Molecular Basis of Nonhost Resistance?. Mol. Plant Microbe Interact..

[28] Toruño T.Y., Stergiopoulos I., Coaker G. (2016). Plant-Pathogen Effectors: Cellular Probes Interfering with Plant Defenses in Spatial and Temporal Manners. Annu. Rev. Phytopathol..

[29] Vleeshouwers V.G., Rietman H., Krenek P., Champouret N., Young C., Oh S.K., Wang M., Bouwmeester K., Vosman B., Visser R.G. (2008). Effector genomics accelerates discovery and functional profiling of potato disease resistance and phytophthora infestans avirulence genes. PLoS ONE.

[30] Lewis J.D., Abada W., Ma W., Guttman D.S., Desveaux D. (2008). The HopZ family of Pseudomonas syringae type III effectors require myristoylation for virulence and avirulence functions in *Arabidopsis thaliana*. J. Bacteriol..

[31] Jayaraman J., Choi S., Prokchorchik M., Choi D.S., Spiandore A., Rikkerink E.H., Templeton M.D., Segonzac C., Sohn K.H. (2017). A bacterial acetyltransferase triggers immunity in *Arabidopsis thaliana* independent of hypersensitive response. Sci. Rep..

[32] Vinatzer B.A., Teitzel G.M., Lee M.W., Jelenska J., Hotton S., Fairfax K., Jenrette J., Greenberg J.T. (2006). The type III effector repertoire of *Pseudomonas syringae* pv. syringae B728a and its role in survival and disease on host and non-host plants. Mol. Microbiol..

[33] Olawole O.I., Liu Q., Chen C., Gleason M.L., Beattie G.A. (2021). The Contributions to Virulence of the Effectors Eop1 and DspE Differ Between Two Clades of *Erwinia tracheiphila* Strains. Mol. Plant Microbe Interact..

[34] Ma W., Dong F.F., Stavrinides J., Guttman D.S. (2006). Type III effector diversification via both pathoadaptation and horizontal transfer in response to a coevolutionary arms race. PLoS Genet..

[35] Nazareno E.S., Kersey C.M., Dumenyo C.K. (2016). Characterization of the incompatible interaction between Erwinia tracheiphila and non-host tobacco (*Nicotiana tabacum*). Physiol. Mol. Plant Pathol..

[36] Zhang Z.M., Ma K.W., Gao L., Hu Z., Schwizer S., Ma W., Song J. (2017). Mechanism of host substrate acetylation by a YopJ family effector. Nat. Plants.

[37] Polgár L. (2005). The catalytic triad of serine peptidases. Cell Mol. Life Sci..

[38] Catalano C., Al Mughram M.H., Guo Y., Kellogg G.E. (2021). 3D interaction homology: Hydropathic interaction environments of serine and cysteine are strikingly different and their roles adapt in membrane proteins. Curr. Res. Struct. Biol..

[39] Ballinger P., Long F.A. (1960). Acid Ionization Constants of Alcohols. II. Acidities of Some Substituted Methanols and Related Compounds1,2. J. Am. Chem. Soc..

[40] Gisdon F.J., Bombarda E., Ullmann G.M. (2022). Serine and Cysteine Peptidases: So Similar, Yet Different. How the Active-Site Electrostatics Facilitates Different Reaction Mechanisms. J. Phys. Chem. B.

[41] Nozaki Y., Tanford C. (1967). [84] Examination of titration behavior. Methods in Enzymology.

[42] Thurlkill R.L., Grimsley G.R., Scholtz J.M., Pace C.N. (2006). pK values of the ionizable groups of proteins. Protein Sci..

[43] Patel S. (2017). A critical review on serine protease: Key immune manipulator and pathology mediator. Allergol. Immunopathol..

[44] Warshel A., Naray-Szabo G., Sussman F., Hwang J.K. (1989). How do serine proteases really work?. Biochemistry.

[45] Prah A., Frančišković E., Mavri J., Stare J. (2019). Electrostatics as the Driving Force Behind the Catalytic Function of the Monoamine Oxidase A Enzyme Confirmed by Quantum Computations. ACS Catal..

[46] Mladenovic M., Fink R.F., Thiel W., Schirmeister T., Engels B. (2008). On the origin of the stabilization of the zwitterionic resting state of cysteine proteases: A theoretical study. J. Am. Chem. Soc..

[47] Ishida T., Kato S. (2003). Theoretical perspectives on the reaction mechanism of serine proteases: The reaction free energy profiles of the acylation process. J. Am. Chem. Soc..

[48] Beveridge A.J. (1996). A theoretical study of the active sites of papain and S195C rat trypsin: Implications for the low reactivity of mutant serine proteinases. Protein Sci..

[49] Dardenne L.E., Werneck A.S., de Oliveira Neto M., Bisch P.M. (2003). Electrostatic properties in the catalytic site of papain: A possible regulatory mechanism for the reactivity of the ion pair. Proteins.

[50] Asadi M., Oanca G., Warshel A. (2022). Effect of Environmental Factors on the Catalytic Activity of Intramembrane Serine Protease. J. Am. Chem. Soc..

[51] Madeira F., Park Y.M., Lee J., Buso N., Gur T., Madhusoodanan N., Basutkar P., Tivey A.R.N., Potter S.C., Finn R.D. (2019). The EMBL-EBI search and sequence analysis tools APIs in 2019. Nucleic Acids Res..

[52] Jumper J., Evans R., Pritzel A., Green T., Figurnov M., Ronneberger O., Tunyasuvunakool K., Bates R., Žídek A., Potapenko A. (2021). Highly accurate protein structure prediction with AlphaFold. Nature.

[53] Park D.H., Yu J.G., Oh E.J., Han K.S., Yea M.C., Lee S.J., Myung I.S., Shim H.S., Oh C.S. (2016). First Report of Fire Blight Disease on Asian Pear Caused by *Erwinia amylovora* in Korea. Plant Dis..

[54] Jeleńska J., Lee J., Manning A.J., Wolfgeher D.J., Ahn Y., Walters-Marrah G., Lopez I.E., Garcia L., McClerklin S.A., Michelmore R.W. (2021). *Pseudomonas syringae* effector HopZ3 suppresses the bacterial AvrPto1-tomato PTO immune complex via acetylation. PLoS Pathog..

[55] Kawai T., Akira S. (2010). The role of pattern-recognition receptors in innate immunity: Update on Toll-like receptors. Nat. Immunol..

[56] Asselin J.E., Bonasera J.M., Kim J.F., Oh C.S., Beer S.V. (2011). Eop1 from a Rubus strain of Erwinia amylovora functions as a host-range limiting factor. Phytopathology.

[57] Cheng Y., Oldfield C.J., Meng J., Romero P., Uversky V.N., Dunker A.K. (2007). Mining α-Helix-Forming Molecular Recognition Features with Cross Species Sequence Alignments. Biochemistry.

[58] Disfani F.M., Hsu W.-L., Mizianty M.J., Oldfield C.J., Xue B., Dunker A.K., Kurgan L. (2012). MoRFpred, a computational tool for sequence-based prediction and characterization of short disorder-to-order transitioning binding regions in proteins. Bioinformatics.

[59] Kim W.S., Jock S., Paulin J.P., Rhim S.L., Geider K. (2001). Molecular Detection and Differentiation of Erwinia pyrifoliae and Host Range Analysis of the Asian Pear Pathogen. Plant Dis..

[60] Kube M., Migdoll A.M., Muller I., Kuhl H., Beck A., Reinhardt R., Geider K. (2008). The genome of Erwinia tasmaniensis strain Et1/99, a non-pathogenic bacterium in the genus Erwinia. Environ. Microbiol..

[61] Lee G.M., Ko S., Oh E.J., Song Y.R., Kim D., Oh C.S. (2020). Comparative Genome Analysis Reveals Natural Variations in the Genomes of Erwinia pyrifoliae, a Black Shoot Blight Pathogen in Apple and Pear. Plant Pathol. J..

[62] Rojas E.S., Batzer J.C., Beattie G.A., Fleischer S.J., Shapiro L.R., Williams M.A., Gleason M.L. (2015). Bacterial Wilt of Cucurbits: Resurrecting a Classic Pathosystem. Plant Dis..

[63] Shapiro L.R., Paulson J.N., Arnold B.J., Scully E.D., Zhaxybayeva O., Pierce N.E., Kolter R. (2018). An Introduced Crop Plant Is Driving Diversification of the Virulent Bacterial Pathogen Erwinia tracheiphila. mBio.

[64] Walterson A.M., Stavrinides J. (2015). Pantoea: Insights into a highly versatile and diverse genus within the Enterobacteriaceae. FEMS Microbiol. Rev..

