# Supplementary material for: Structure-Function Characterisation of Eop1 Effectors from the Erwinia-Pantoea Clade Reveals They May Acetylate Their Defence Target through a Catalytic Dyad
Source: Int J Mol Sci. 2023 Sep 28;24(19):14664. doi: 10.3390/ijms241914664 (PMC10572645; doi:10.3390/ijms241914664)
Supplement: Supplementary file 1 [file ijms-24-14664-s001.zip › ijms-2602796-supplementary.pdf]

SUPPLEMENTARY MATERIAL

	HopZ3psy	Ep1/96	Ea246	Ea262	Et1/99	E.tr_MDcuke	P. va_C9-1
HopZ3psy		54.8%	55.0%	52.3%	54.3%	48.7%	48.2%
Ep1/96	54.8%		68.7%	80.6%	70.4%	55.6%	58.8%
Ea246	55.0%	68.7%		66.8%	82.4%	56.5%	61.9%
Ea262	52.3%	80.6%	66.8%		69.1%	55.5%	57.7%
Et1/99	54.3%	70.4%	82.4%	69.1%		56.5%	61.2%
E.tr_MDcuke	48.7%	55.6%	56.5%	55.5%	56.5%		69.2%
P. va_C9-1	48.2%	58.8%	61.9%	57.7%	61.2%	69.2%	

Supplementary Figure S1: Protein sequence alignment matrix presenting the percentage similarity between the protein sequences of *Erwinia-Pantoea* clade Eop1 variants tested in the HR assay.

Supplementary Table S1: An overview of the essential details of the Eop1 variants selected for the HR assay in *Nicotiana tabacum*.

**In the current study, referred as**	**Accession No. in NCBI database**	**Resident species of the Eop1 variant**	**Associated host plants**
Ep1/96	WP_012669297.1	*Erwinia pyrifoliae* str. Ep1/96	Pathogenic to Asian pear (*Pyrus pyrifoliae)* but also infects some cultivars of apple (Kim et al., 2001; Lee et al., 2020).
Ea246	AAF63400.1	*Erwinia amylovora* str. Ea246	*Rubus*-infecting strains of *E. amylovora*, particularly raspberry and blackberry (Asselin et al., 2011).
Ea262	AEH03408.1	*Erwinia amylovora* str. Ea262	Spiraeoideae-infecting strain of *E. amylovora,* particularly apple and pear (Asselin et al., 2011).
Et1/99	WP_012440293.1	*Erwinia tasmaniensis* str. Et1/99	An epiphytic and a putative non-phytopathogenic relative of *E. amylovora* (Kube et al., 2008).
E. tr_MDcuke	AXF77196.1	*E. tracheiphila* str. MDcuke	The etiological agent of the bacterial wilt disease in Cucurbitaceae, predominantly infecting *Cucurbita* and *Cucumis* species. Localised to the Midwest, Mid-Atlantic, and Northeast regions of the USA, along with southern portions of Canada (Rojas et al., 2015; Shapiro et al., 2018).
P. va_C9-1	WP_061060943.1	*Pantoea vagans* str. C9-1	*P. vagans* (formerly *Erwinia herbicola*) is a non-phytopathogenic epiphyte species, used as an antagonistic biocontrol agent against *E. amylovora* (Walterson & Stavrinides, 2015).

Supplementary Table S2: Sequence length of the regulatory and catalytic domains in the protein sequence of the *Erwinia-Pantoea* clade Eop1 variants, HopZ1a and HopZ3psa

Effector	Seq. length of the N-terminus region	Regulatory domain 1 Seq. length	Catalytic domain Seq. length	Regulatory domain 2 Seq. length	References
HopZ1a	1–45	46–83	84–284	285–369	(Lewis et al., 2008; Zhang et al., 2016)
Ep1/96	1–127	128–164	165–324	325–401	This study
Ea246	1–120	121–157	158–316	317–393	This study
Ea262	1–127	128–164	165–324	325–401	This study
Et1/99	1–123	124–160	161–319	320–396	This study
E. tr_MDcuke	1–135	136–172	173–332	333–409	This study
P.va_C9-1	1–133	134–170	171–330	331–407	This study
HopZ3$_{psy}$	1–133	134–170	171–330	331–407	(Lewis et al., 2008), this study (AlphaFold prediction)
Note: The sequence length of the regulatory and catalytic domains in the effectors mentioned above is predicted based on the protein sequence alignment with HopZ1a via Clustal Omega. The catalytic triad residue sites for the same effectors are mentioned in Supplementary Table 3.					

Supplementary Table S3**:** Predicted catalytic triad residue sites in the tested Eop1 variants and compared with HopZ1a.

**YopJ Effector**	**Catalytic histidine site in the sequence**	**Catalytic glutamate site in the sequence**	**Catalytic cysteine site in the sequence**	**References**
HopZ1a	150	170	216	(Lewis et al., 2008; Zhang et al., 2016)
Ep1/96	235	255	292	This study
Ea246	228	248	285	This study
Ea262	235	255	292	This study
Et1/99	231	251	288	This study
E. tr_MDcuke	243	263	300	This study
P. va_C9-1	241	261	298	This study
HopZ3$_{psy}$	241	261	298	This study

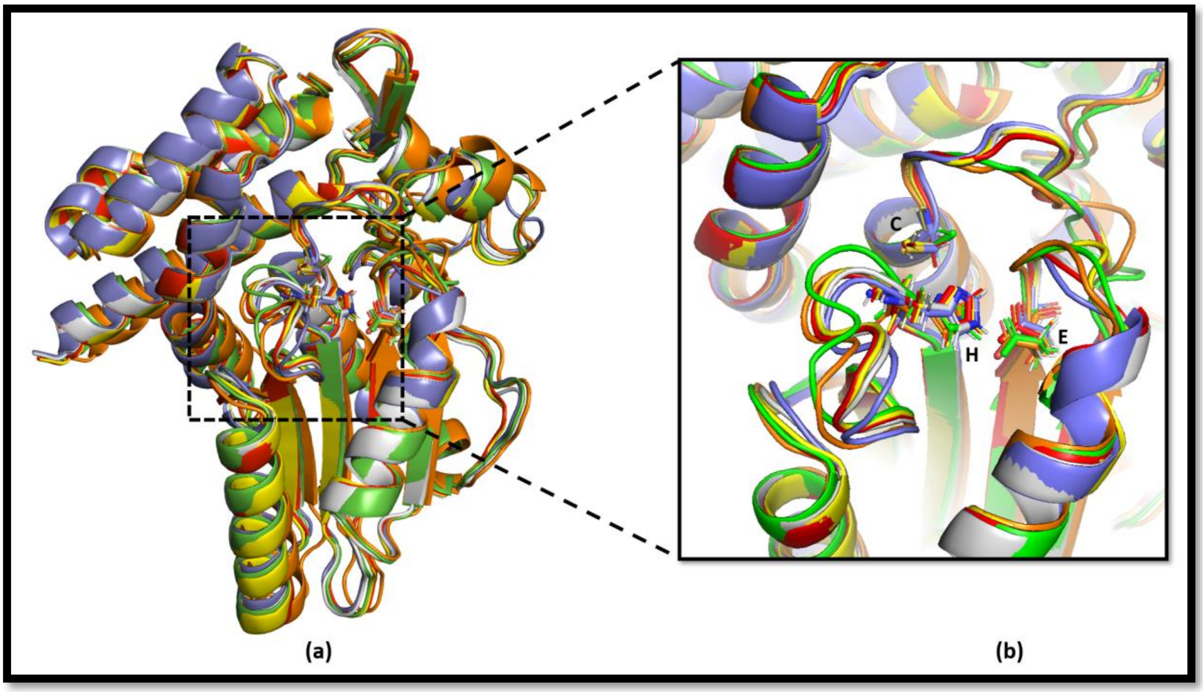

Supplementary Figure S2: *Erwinia-Pantoea* clade Eop1 variant AlphaFold 2 models superimposition analysis. (a) Superimposition of the *Erwinia-Pantoea* clade Eop1 variants' tertiary structure. (b) Superimposition of the Eop1 variants' catalytic triad. The catalytic triad residues are annotated as, H = histidine, E = glutamic acid, and C = cysteine. The colour codes are as follows: red = *Ea*246 (WT), green = *Ep*1/96, yellow = *Et*1/99, grey = *P. va*_C9-1, orange = *Ea*262, blue = *E. tr*_MDcuke.

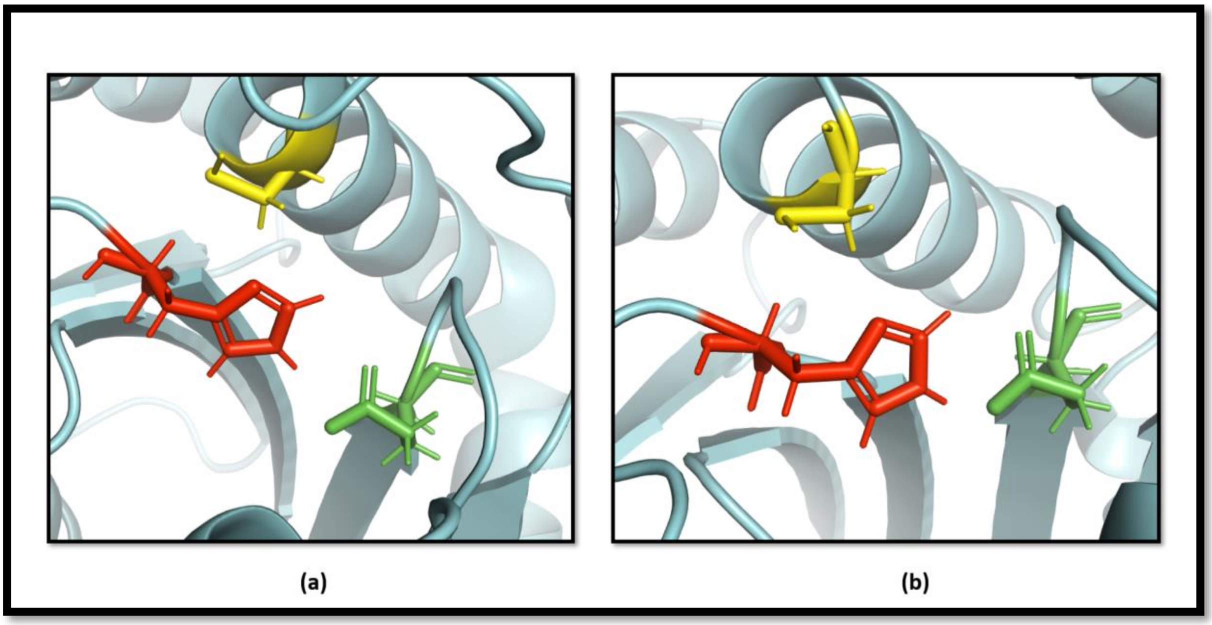

Supplementary Figure S3: Analysis of the structural and 3D conformation homology between HopZ1a and Ea246 Eop1 catalytic triad (magnified view of the catalytic pocket). (a) HopZ1a catalytic triad. (b) Ea246 Eop1 catalytic triad. Catalytic triad residues, histidine (H), glutamic acid (E), and cysteine (C), are coloured red, green, and yellow respectively.

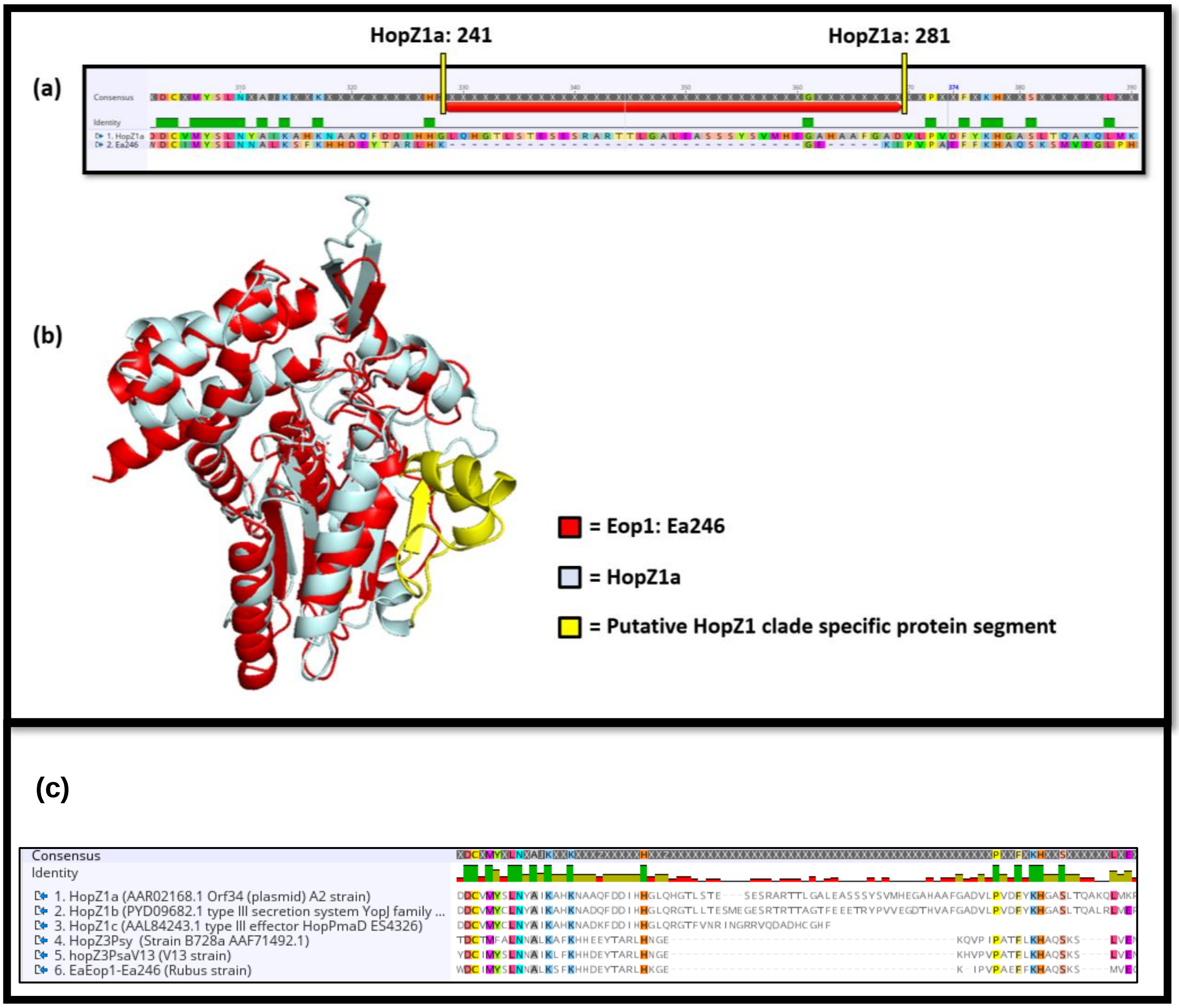

Supplementary Figure S4: Identification of a putative HopZ1 clade-specific protein segment via superimposition analysis with an *Erwinia-Pantoea* clade Eop1: Ea246. (a) Protein sequence alignment of HopZ1a (AAR02168) with *Ea*246 (AAF63400); the red bar below the consensus corresponds to the sequence length of the identified HopZ1 clade-specific protein segment. (b) Tertiary structure superimposition analysis of HopZ1a and Ea246 Eop1. The colour codes are presented on the bottom-right of the image. (c) Alignment of HopZ effectors in the HopZ1a specific region showing significant variation in length and sequence.

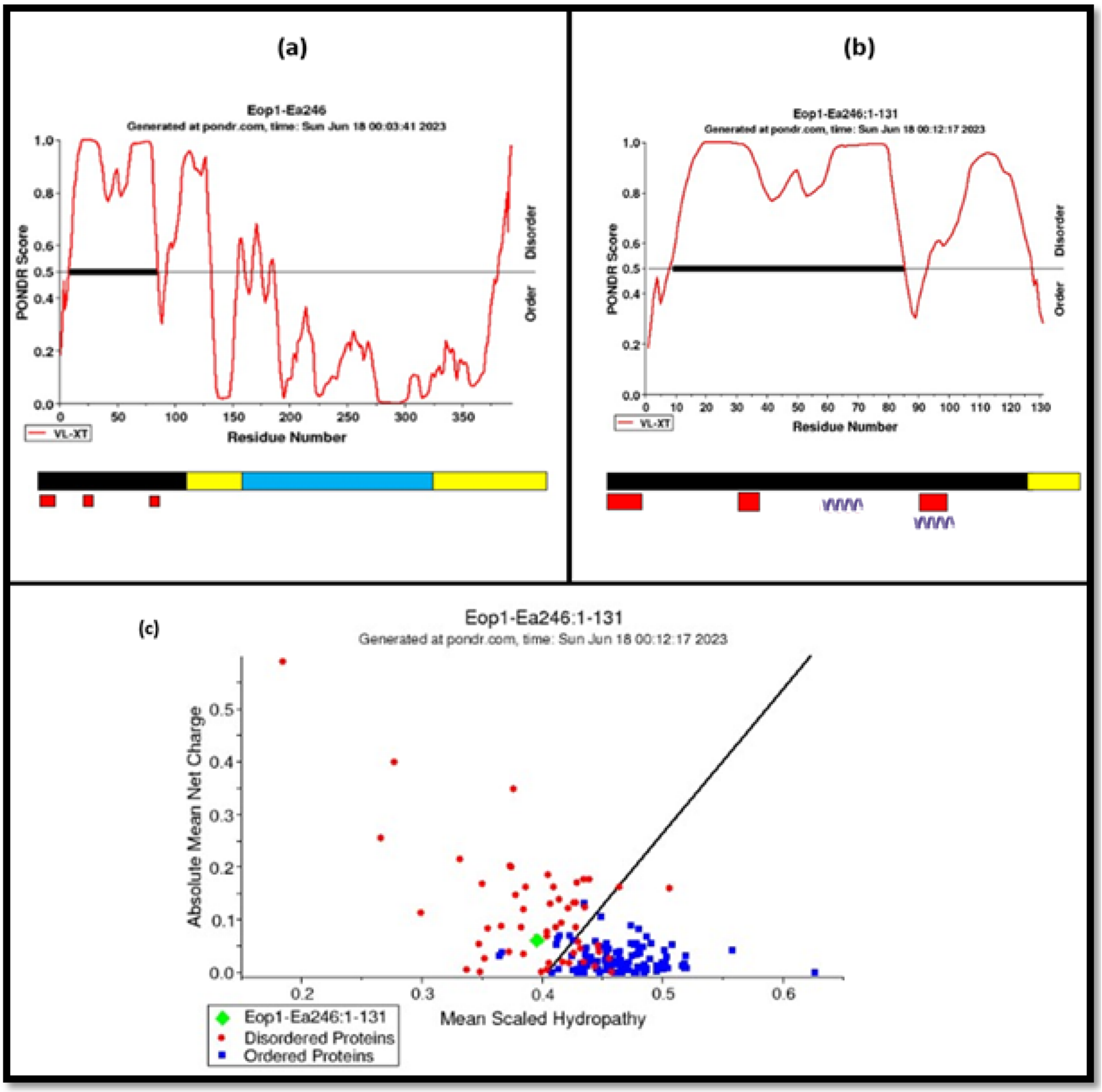

Supplementary Figure S5: Analysis of the intrinsically disordered protein regions in Eop1-Ea246 effector via PONDR plots. (a) VL-XT (Cheng et al., 2007) score of full length Eop1 with the disordered N-terminal region (black rectangle), two structured regulatory regions (yellow rectangles) and the structured catalytic region (blue rectangle) and the likely Molecular Recognition regions (MoRFs; red rectangles) annotated below. (b) PONDR score analysis of the disordered N-terminal region of Eop1-Ea246 with the disordered N-terminal region, the start of the first structured regulatory region, the MoRFs predicted by MoRFpred (Disfani et al., 2012) and two alpha helices (purple) annotated below. (c) Charge hydropathy plot of the first 131 residues containing the disordered N-terminal region of Eop1-Ea246 compared with the respective positions of a database of ordered (blue) and disordered (red) proteins and a black “divider line” between predicted ordered and disordered states.

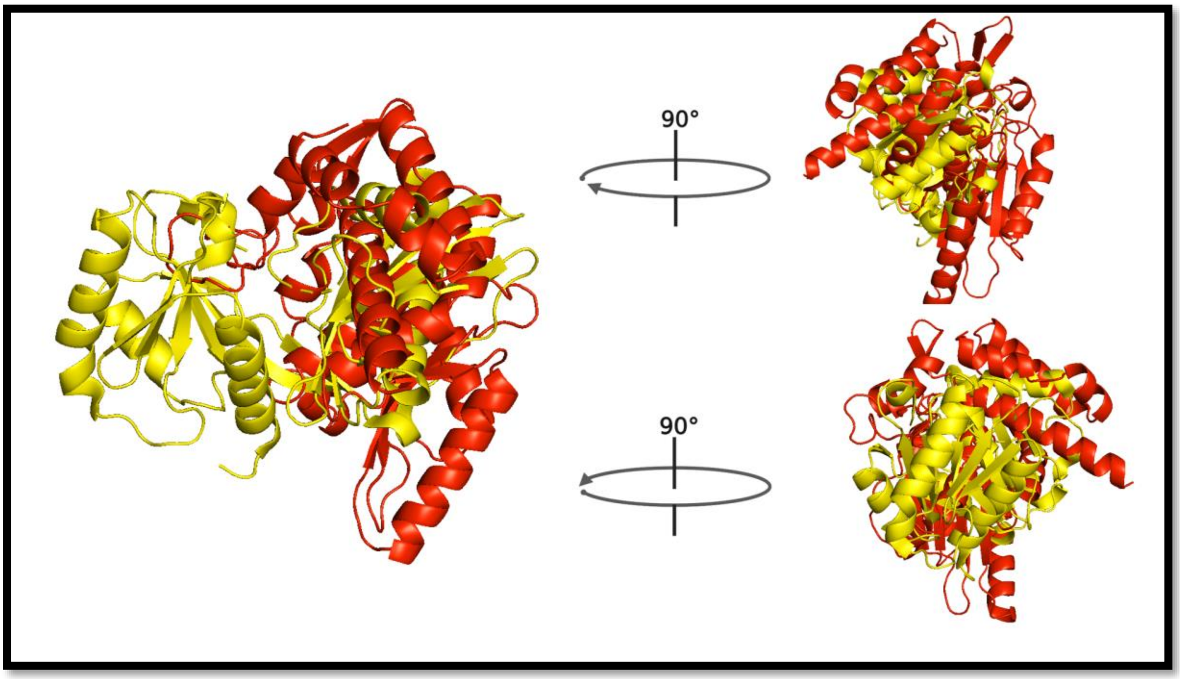

Supplementary Figure S6: Ea246 (red) and GCN5-related N-acetyltransferase (yellow; PDB: 4IUS) superimposition analysis.

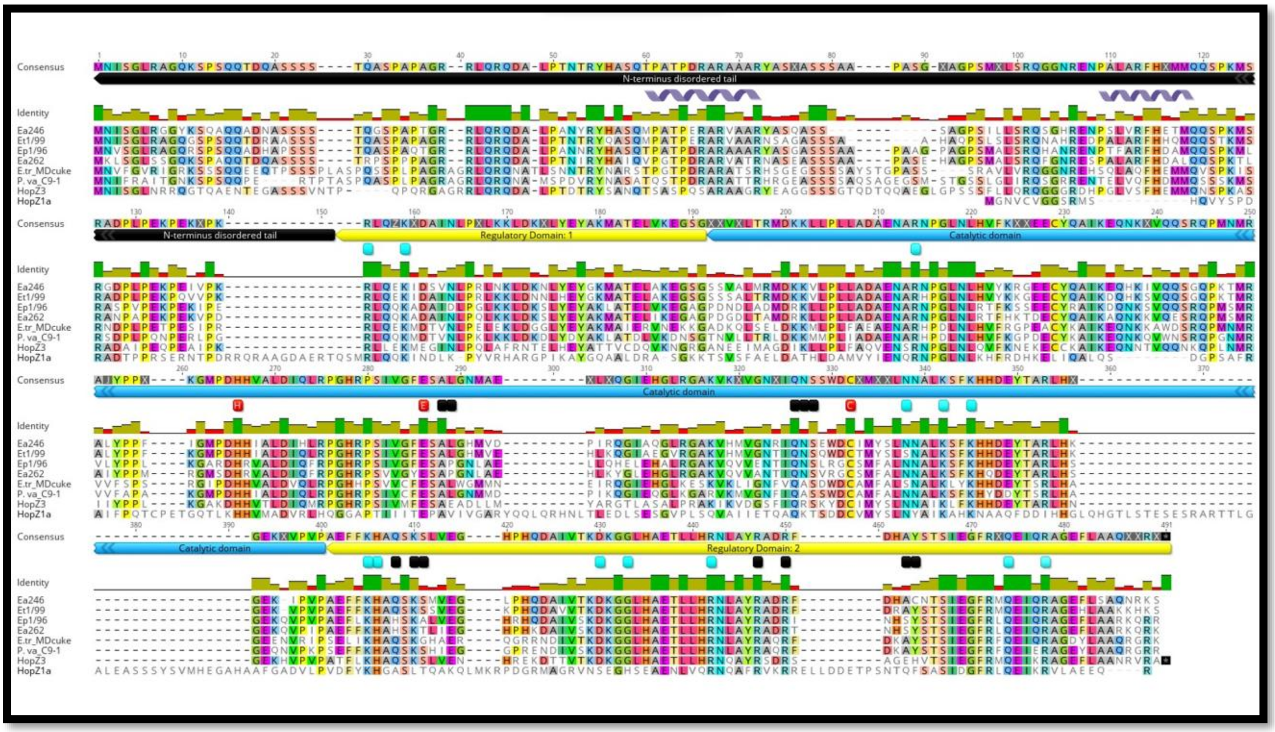

Supplementary Figure S7: Protein sequence alignment of *Erwinia-Pantoea* clade and HopZ YopJ variants. The amino acid sequences were aligned using Clustal Omega via Geneious 2021. The bars below the consensus represent regulatory (yellow) and catalytic (sky-blue) domains' length in the proteins. The black and cyan colour squares below the consensus and above the identity graph correspond to the AcCoA-interacting and IP6-interacting residues, respectively. The catalytic triad residues, histidine, glutamic acid, and cysteine, are presented in the red squares. Two alpha helices in the largely disordered N-terminal region, predicted by AlphaFold2 for Eop1 variants and HopZ3$_{Psy}$ (not HopZ1a), are depicted via purple helices above the identity graph.

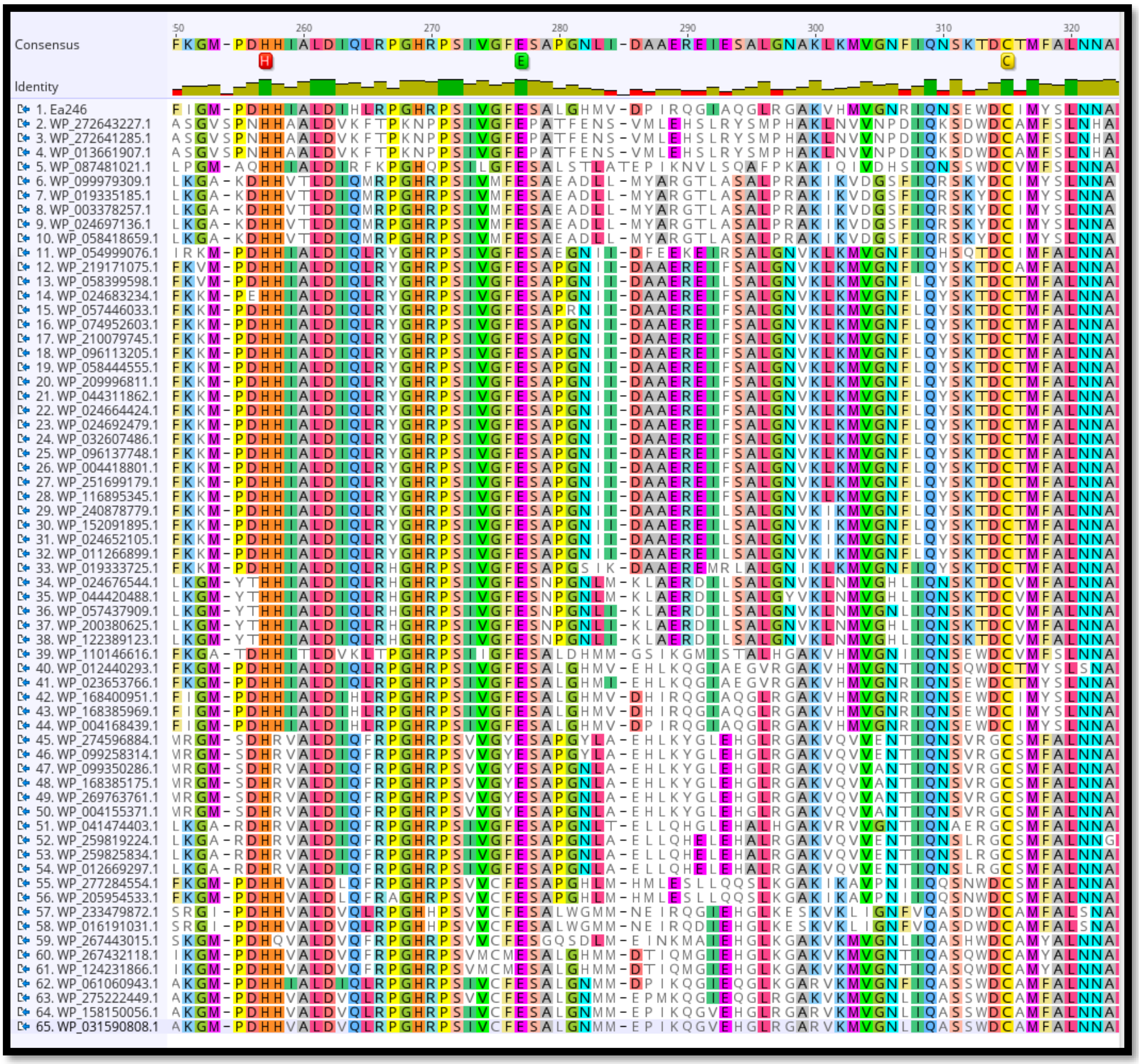

Supplementary Figure S8: Broad-scale conservation analysis of the catalytic triad residues: H/E/C in the Eop1-like protein sequences. The protein sequences were obtained via a BLASTp search with Ea246 Eop1 protein sequence used as a query sequence. The sequences with a 'query cover' of 75 % and above were selected for the analysis. The catalytic triad residues, histidine (H), glutamic acid (E), and cysteine (C), are colour-coded in red, green, and yellow boxes, respectively. Residue S249 (numbering based on Eop1Ea246) is adjacent to the putative catalytic triad 248E, while residues S281 and S289 bracket the putative catalytic triad 285C.

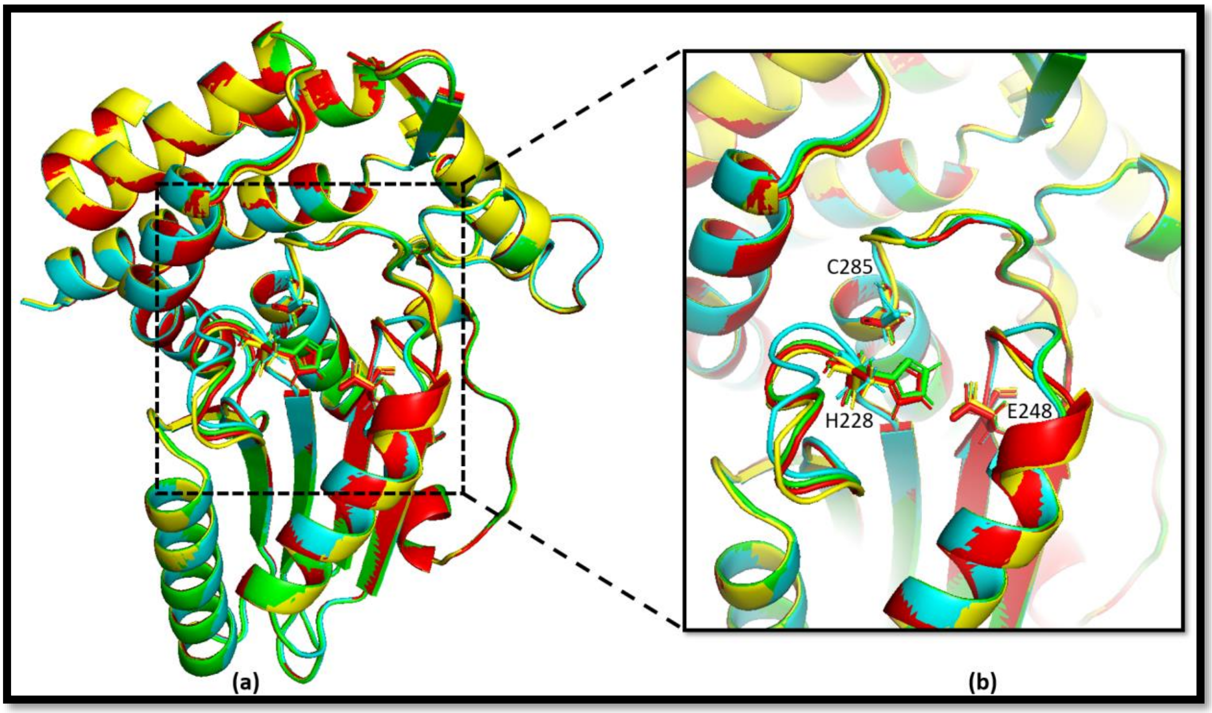

Supplementary Figure S9: Superimposition analysis of AlphaFold2 predicted tertiary structures of Ea246 catalytic triad mutants onto Ea246 (wildtype). (a) superimposition of the predicted tertiary structure of Ea246 Eop1 and its catalytic triad mutants. (b) magnification of the catalytic motif in ‘a’ with the catalytic triad residue positions annotated. Tertiary structures are colour coded, red = Ea246 (WT), green = Ea246: C285A, cyan = Ea246: H228A, Yellow = Ea246_E248A.

Supplementary Table S4: A list of the primer pairs used to generate mutations in the Eop1: Ea246 backbone.

S. No	Ea246 mutation sites	Primer type	Primer pair's sequence
1.	H228A	Forward	5'—ATCCCCCTTTTATAGGCATGCCTGAC**GC**CCATATCGCCCTGGACATTCATC—3'
		Reverse	3'—GATGAATGTCCAGGGCGATATGG**GC**GTCAGGCATGCCTATAAAAGGGGGAT—5. '
2.	C285A	Forward	5'—CAGGATTCAAAATTCCGAGTGGGAC**GC**CATCATGTACTCGTTGAACAATGCCT—3'
		Reverse	3'—AGGCATTGTTCAACGAGTACATGATG**GC**GTCCCACTCGGAATTTTGAATCCTG—5'
3.	C285S	Forward	5'—CAGGATTCAAAATTCCGAGTGGGACT**C**CATCATGTACTCGTTGAACAATGCCT—3'
		Reverse	3'—AGGCATTGTTCAACGAGTACATGATG**G**AGTCCCACTCGGAATTTTGAATCCTG—5'
4.	S281A	Forward	5'—TATGGTAGGCAACAGGATTCAAAAT**G**CCGAGTGGGACTGCATCATGTACTC—3'
		Reverse	3'—GAGTACATGATGCAGTCCCACT**C**GGCATTTTGAATCCTGTTGCCTACCATA—5'
5.	S281A + C285A	Forward	5'—TATGGTAGGCAACAGGATTCAAAAT**G**CCGAGTGGGAC**GC**CATCATGTACTC—3'
		Reverse	3'—GAGTACATGATG**GC**GTCCCACT**C**GGCATTTTGAATCCTGTTGCCTACCATA—5'
6.	E248A	-	Synthesised
7.	S249A	-	Synthesised
8.	H228A + C2585A	-	The double mutation was produced in the Eop1_Ea246 backbone using the H228A primer pair with an entry vector carrying the C285A mutation.

Note: The bold and highlighted letters in the PCR primer sequences represent the introduced mutations. The mutation sites marked as **'synthesised'** were artificially synthesised in the pTwist-ENTR cloning vector via Twist technology [South San Francisco, California, USA] in the Ea246 backbone. All the mutations were verified by M13-pUC primers-driven sequencing. Attempts to clone an S289A mutation via PCR were not successful, so, the primer sequence for the same are not given and this mutant was not tested.

SUPPLEMENTARY REFERENCES

Asselin, J. E., Bonasera, J. M., Kim, J. F., Oh, C. S., & Beer, S. V. (2011). Eop1 from a *Rubus* strain of *Erwinia amylovora* functions as a host-range limiting factor. *Phytopathology*, *101*(8), 935-944. https://doi.org/10.1094/PHYTO-12-10-0339

Cheng, Y., Oldfield, C. J., Meng, J., Romero, P., Uversky, V. N., & Dunker, A. K. (2007). Mining α-Helix-Forming Molecular Recognition Features with Cross Species Sequence Alignments. *Biochemistry*, *46*(47), 13468-13477. https://doi.org/10.1021/bi7012273

Disfani, F. M., Hsu, W.-L., Mizianty, M. J., Oldfield, C. J., Xue, B., Dunker, A. K., . . . Kurgan, L. (2012). MoRFpred, a computational tool for sequence-based prediction and characterization of short disorder-to-order transitioning binding regions in proteins. *Bioinformatics*, *28*(12), i75-i83. https://doi.org/10.1093/bioinformatics/bts209

Kim, W. S., Jock, S., Paulin, J. P., Rhim, S. L., & Geider, K. (2001). Molecular Detection and Differentiation of Erwinia pyrifoliae and Host Range Analysis of the Asian Pear Pathogen. *Plant Dis*, *85*(11), 1183-1188. https://doi.org/10.1094/PDIS.2001.85.11.1183

Kube, M., Migdoll, A. M., Muller, I., Kuhl, H., Beck, A., Reinhardt, R., & Geider, K. (2008). The genome of Erwinia tasmaniensis strain Et1/99, a non-pathogenic bacterium in the genus Erwinia. *Environ Microbiol*, *10*(9), 2211-2222. https://doi.org/10.1111/j.1462-2920.2008.01639.x

Lee, G. M., Ko, S., Oh, E. J., Song, Y. R., Kim, D., & Oh, C. S. (2020). Comparative Genome Analysis Reveals Natural Variations in the Genomes of Erwinia pyrifoliae, a Black Shoot Blight Pathogen in Apple and Pear. *Plant Pathol J*, *36*(5), 428-439. https://doi.org/10.5423/PPJ.OA.06.2020.0097

Lewis, J. D., Abada, W., Ma, W., Guttman, D. S., & Desveaux, D. (2008). The HopZ family of Pseudomonas syringae type III effectors require myristoylation for virulence and avirulence functions in Arabidopsis thaliana. *J Bacteriol*, *190*(8), 2880-2891. https://doi.org/10.1128/jb.01702-07

Rojas, E. S., Batzer, J. C., Beattie, G. A., Fleischer, S. J., Shapiro, L. R., Williams, M. A., . . . Gleason, M. L. (2015). Bacterial Wilt of Cucurbits: Resurrecting a Classic Pathosystem. *Plant Dis*, *99*(5), 564-574. https://doi.org/10.1094/PDIS-10-14-1068-FE

Shapiro, L. R., Paulson, J. N., Arnold, B. J., Scully, E. D., Zhaxybayeva, O., Pierce, N. E., . . . Kolter, R. (2018). An Introduced Crop Plant Is Driving Diversification of the Virulent Bacterial Pathogen Erwinia tracheiphila. *mBio*, *9*(5), e01307-01318. https://doi.org/10.1128/mBio.01307-18

Walterson, A. M., & Stavrinides, J. (2015). Pantoea: insights into a highly versatile and diverse genus within the Enterobacteriaceae. *FEMS Microbiol Rev*, *39*(6), 968-984. https://doi.org/10.1093/femsre/fuv027

Zhang, Z. M., Ma, K. W., Yuan, S., Luo, Y., Jiang, S., Hawara, E., . . . Song, J. (2016). Structure of a pathogen effector reveals the enzymatic mechanism of a novel acetyltransferase family. *Nat Struct Mol Biol*, *23*(9), 847-852. https://doi.org/10.1038/nsmb.3279
